# Long-term Neural Embedding of Childhood Adversity in a Population-Representative Birth Cohort Followed for 5 Decades

**DOI:** 10.1016/j.biopsych.2021.02.971

**Published:** 2021-08-01

**Authors:** Maria Z. Gehred, Annchen R. Knodt, Antony Ambler, Kyle J. Bourassa, Andrea Danese, Maxwell L. Elliott, Sean Hogan, David Ireland, Richie Poulton, Sandhya Ramrakha, Aaron Reuben, Maria L. Sison, Terrie E. Moffitt, Ahmad R. Hariri, Avshalom Caspi

**Affiliations:** a Department of Psychology & Neuroscience, Duke University, Durham, North Carolina; b Center for Genomic and Computational Biology, Duke University, Durham, North Carolina; c Department of Psychiatry & Behavioral Sciences, Duke University School of Medicine, Durham, North Carolina; d Dunedin Multidisciplinary Health and Development Research Unit, Department of Psychology, University of Otago, Dunedin, New Zealand; e Social, Genetic, and Developmental Psychiatry Research Center, Institute of Psychiatry, Psychology & Neuroscience, King’s College London, London, United Kingdom; f Department of Child and Adolescent Psychiatry, Institute of Psychiatry, Psychology & Neuroscience, King’s College London, London, United Kingdom; g National and Specialist Child and Adolescent Mental Health Services Clinic for Trauma, Anxiety and Depression, South London and Maudsley National Health Service Foundation Trust, London, United Kingdom; h PROMENTA Center, University of Oslo, Norway

**Keywords:** Childhood adversity, Maltreatment, Midlife brain structure, Prospective, Retrospective

## Abstract

**Background:**

Childhood adversity has been previously associated with alterations in brain structure, but heterogeneous designs, methods, and measures have contributed to mixed results and have impeded progress in mapping the biological embedding of childhood adversity. We sought to identify long-term differences in structural brain integrity associated with childhood adversity.

**Methods:**

Multiple regression was used to test associations between prospectively ascertained adversity during childhood and adversity retrospectively reported in adulthood with structural magnetic resonance imaging measures of midlife global and regional cortical thickness, cortical surface area, and subcortical gray matter volume in 861 (425 female) members of the Dunedin Study, a longitudinal investigation of a population-representative birth cohort.

**Results:**

Both prospectively ascertained childhood adversity and retrospectively reported adversity were associated with alterations in midlife structural brain integrity, but associations with prospectively ascertained childhood adversity were consistently stronger and more widely distributed than associations with retrospectively reported childhood adversity. Sensitivity analyses revealed that these associations were not driven by any particular adversity or category of adversity (i.e., threat or deprivation) or by childhood socioeconomic disadvantage. Network enrichment analyses revealed that these associations were not localized but were broadly distributed along a hierarchical cortical gradient of information processing.

**Conclusions:**

Exposure to childhood adversity broadly is associated with widespread differences in midlife gray matter across cortical and subcortical structures, suggesting that biological embedding of childhood adversity in the brain is long lasting, but not localized. Research using retrospectively reported adversity likely underestimates the magnitude of these associations. These findings may inform future research investigating mechanisms through which adversity becomes embedded in the brain and influences mental health and cognition.

SEE COMMENTARY ON PAGE 143

Childhood adversity, including abuse, neglect, and family disruption, is associated with lasting negative health outcomes. A growing body of research documents consistent dose-response associations between childhood adversity and later psychopathology (1, 2, 3, 4, 5, 6, 7). Identifying mechanisms through which adversity becomes biologically embedded and thereby disrupts development has been a vigorous focus of neuropsychiatric research (8, 9, 10, 11, 12, 13). To date, research has revealed that childhood adversity is associated with alterations in the structural integrity of affective, motivational, mnemonic, and executive control networks (7,14, 15, 16, 17, 18, 19, 20, 21, 22, 23, 24, 25, 26, 27, 28, 29, 30, 31, 32, 33). However, the existing evidence for such biological embedding of adversity is limited in several ways.

First, the majority of studies have focused on a small number of a priori regions of interest, most prominently the amygdala and hippocampus. This targeted approach, at least initially, reflected results from animal models demonstrating sensitivity of these brain regions to chronic stress (15). Even with such a targeted approach, the findings have been inconsistent. For example, in child or adolescent samples scanned closer to the exposure, adversity has been associated with fewer, more, and no differences in amygdala gray matter volume (20, 21, 22, 23, 24). Although region-of-interest approaches predominate, whole-brain studies have reported associations between childhood adversity and gray matter volume of regions extending beyond the amygdala and hippocampus, including the thalamus and insula (17), and have moved beyond volumetric measures of cortex to more fine-grained measures of surface area and thickness. Findings from these studies, however, have also been mixed. While most studies of children and adults report null, or negative, associations between childhood adversity and surface area or cortical thickness of prefrontal, parietal, or temporal regions [e.g., (25, 26, 27)], a recent study reported positive associations between childhood institutionalization and cortical surface area and thickness of temporal regions in adulthood (28). Perhaps contributing to this inconsistency, there have been few adequately powered whole-brain studies. Regardless, the emerging literature suggests that a continued focus on a small number of regions may lead to a misleadingly localized view of how adverse childhood experiences (ACEs) may become embedded.

Second, most research on biological embedding of childhood adversity relies on cross-sectional designs and retrospective reporting. However, a recent meta-analysis revealed that there is only fair agreement between adversity retrospectively reported in adulthood and adversity prospectively ascertained in childhood (34). Moreover, there is concern that adults’ current mental health may bias retrospective reports of childhood experiences (35, 36, 37). Consistent with this concern, prospectively ascertained adversity is more strongly linked to biomarker-indexed physical health outcomes, whereas retrospectively reported adversity is more strongly linked to interview-assessed mental disorders (38, 39, 40). There is less neuroimaging research following children whose exposure to adversity was ascertained prospectively, and prospective studies have often focused on extreme forms of adversity, such as institutionalization [e.g., (27, 28, 29,31)], which may not generalize to more common forms of adversity. Prospective studies have generated seemingly conflicting findings about the brain correlates of childhood adversity (32) and have not always confirmed findings from retrospective studies (33). These mixed results may be due to studies with insufficient power that fail to detect small effects but that can also overestimate true effect sizes (41).

In this study, we report a series of novel analyses undertaken to test biological embedding of childhood adversity. First, given the heterogeneity of existing findings and the tendency to focus on a priori regions of interest, we report results from whole-brain analyses in which we tested associations between childhood adversity and 2 structural features of the cortex—surface area and thickness—as well as subcortical gray matter volume. The patterns of associations identified using a whole-brain approach may indicate mechanisms that operate within discrete circuits and structures, or broader mechanisms, such as chronic inflammation, that act globally. Distinguishing between cortical surface area and thickness, which are related to cortical volume but are independent and have been associated with genetically and developmentally distinct factors (25,42), may provide further insight as to the mechanisms underlying biological embedding of childhood adversity. Second, given the varied measures of adversity used across studies and their inconsistent mapping onto brain, we compare structural associations identified using both prospectively ascertained and retrospectively reported adversity. Furthermore, we report post hoc analyses for associations between childhood experiences of threat versus deprivation and brain structure. Third, we conduct analyses on longitudinal data from a population-representative birth cohort followed to midlife to determine possible long-term embedding of adversity.

## Methods and Materials

A brief description of the samples and measures is reported below. A full description is provided in the Supplement.

### Study Design and Sample

Participants were members of the Dunedin Study, a longitudinal investigation of health and behavior in a population-representative birth cohort. Study members (*N* = 1037) all were born between April 1972 and March 1973 in Dunedin, New Zealand (43). The cohort represented the full range of socioeconomic status (SES) of the South Island of New Zealand and, as adults, matched the New Zealand National Health and Nutrition Survey on key adult health indicators and same-age citizens in the New Zealand census on educational attainment (44). The cohort was 93% white. Data were available at birth, and assessments were conducted every few years, most recently at age 45, when 94% of living Dunedin Study members participated. The relevant ethics committees approved each phase of the Dunedin Study and all Study members provided written informed consent. Neuroimaging was carried out at age 45 in 93% (*n* = 875) of participating Dunedin Study members, who represented the original cohort on key demographic variables (Figure S1). Fourteen neuroimaging datasets were excluded, yielding 861 for analysis.

### Measures

#### Exposure to Childhood Adversity

We assessed 10 categories of ACEs introduced by the CDC–Kaiser Permanente Adverse Childhood Experiences Study (1): 5 types of child harm (physical abuse, emotional abuse, physical neglect, emotional neglect, and sexual abuse) and 5 types of household dysfunction (incarceration of a family member, household substance abuse, household mental illness, loss of a parent, and household partner violence). The number of these adversities experienced yielded a cumulative ACEs score, coded 0, 1, 2, 3, or 4+. The distribution of ACEs for Dunedin Study members included in the current analyses is presented in Figure S2.

Prospectively ascertained ACEs scores were generated from records gathered during 7 biennial assessments carried out from ages 3 to 15, including 1) social service contacts, 2) structured notes from interviewers, pediatricians, psychometricians, and nurses who assessed Dunedin Study children and their parents, 3) teachers’ notes of concern, and 4) parental questionnaires, as previously described (38). Retrospectively reported ACEs scores were derived from structured interviews conducted with Dunedin Study members at age 38, during which Study members were asked about childhood experiences of physical abuse and neglect, emotional abuse and neglect, sexual abuse, and the 5 ACEs relating to household dysfunction, as previously described (38).

#### Covariates

Perinatal complications, assessed from hospital records and coded as the sum of the number of prenatal, intrapartum, and neonatal complications experienced (45), were included to account for preexisting neurodevelopmental differences. Childhood neurocognitive health, derived from an examination at age 3 that included assessments by a pediatric neurologist, standardized tests of cognitive function, receptive language, and motor skills, and examiners’ ratings of emotional and behavioral regulation (46), was also included to account for preexisting neurodevelopmental differences. Perceived stress, assessed via self-reported (47) stress, coping ability, and controllability of events during the past year, was included to account for potential bias of experiences that occurred closer to the magnetic resonance imaging scan on retrospectively reported ACEs. Associations between these covariates and prospectively ascertained and retrospectively reported ACEs as well as measures of midlife brain structure are reported in Table S1.

#### Age-45 Brain Structure

Structural magnetic resonance imaging data acquisition and processing are detailed in the Supplement. For each Dunedin Study member, total cortical surface area and average cortical thickness were extracted from each of the 360 cortical areas in the Human Connectome Project Multi-Modal Parcellation version 1.0 (48). Regional cortical thickness and surface area measures have previously been demonstrated to have excellent test-retest reliability in the Dunedin Study [mean intraclass correlation coefficients = 0.85 and 0.99, respectively (49)]. Additionally, gray matter volumes were extracted for 10 subcortical structures using the FreeSurfer aseg parcellation (https://surfer.nmr.mgh.harvard.edu/). These regional volumes also have excellent test-retest reliability in the Dunedin Study (mean intraclass correlation coefficient = 0.96). Functional and clinical outcomes associated with brain structure in the Dunedin cohort have been reported elsewhere [e.g. (50, 51, 52)].

### Statistical Analyses

All analyses were conducted in R version 3.6.1 (53). Sex was a covariate in all analyses. Analyses were preregistered (https://sites.google.com/site/moffittcaspiprojects/home/projectlist/gehred_2019) and checked for reproducibility by an independent data analyst, who recreated the code using the manuscript and an unaltered copy of the dataset. To permit comparisons of effect sizes across prospectively ascertained versus retrospectively reported ACEs and across different measures of brain structure, we report standardized regression coefficients and 95% confidence intervals (CIs).

We estimated ordinary least squares regression models to test associations between ACEs and total cortical surface area and average cortical thickness. We first investigated associations of prospectively ascertained and retrospectively reported ACEs with these measures of global brain structure in separate regression models and then together in the same model. Each of these regression models was re-estimated with the inclusion of perinatal complications, childhood neurocognitive health, and perceived adult stress as covariates.

As an extension of our global analyses, we conducted exploratory parcelwise analyses of surface area and cortical thickness with prospectively ascertained and retrospectively reported ACEs for each of the 360 regions comprising the parcellation scheme described above (48). We corrected for multiple comparisons across the 360 tests performed for surface area and cortical thickness independently using a false discovery rate (FDR) procedure (54).

Finally, we tested the association between prospectively ascertained and retrospectively reported ACEs with the mean gray matter volumes of 10 bilateral subcortical structures using ordinary least squares regression models. Mirroring the above analyses, we first investigated prospectively ascertained and retrospectively reported ACEs in separate regression models and then together in the same model. Each of these regression models also was repeated with the inclusion of perinatal complications, childhood neurocognitive health, and perceived adult stress as covariates. Results were FDR corrected for multiple comparisons (54) across the 10 tests performed.

#### Sensitivity Analyses

We conducted several sensitivity analyses to further probe the robustness of associations between prospectively ascertained ACEs and brain structure, revealed in our primary analyses, including differences in total intracranial volume, childhood SES, and specific forms of adversity (Supplement).

#### Network Enrichment Analysis

Considering the widespread cortical differences identified in our primary analyses, we conducted a network enrichment analysis to test whether the parcelwise associations were enriched within specific networks along a cortical gradient of hierarchical information processing (Supplement) (55).

## Results

### Are Prospectively Ascertained and Retrospectively Reported ACEs Associated With Cortical Surface Area and Thickness?

**Figure 1:**
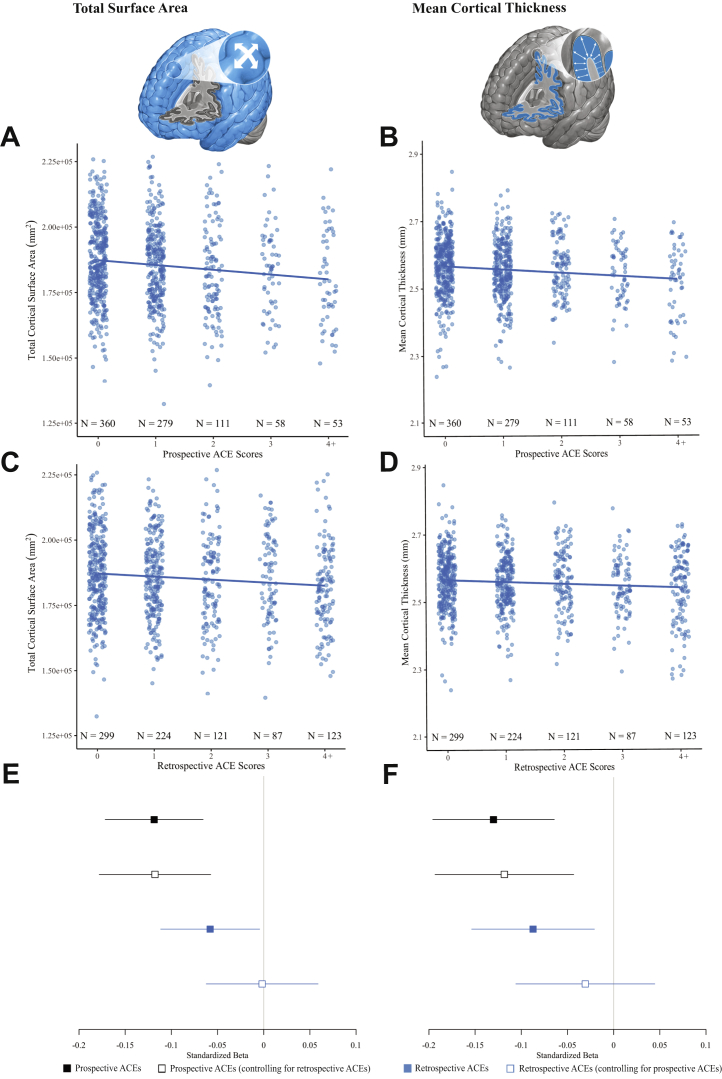
Associations between childhood adversity and global brain structure. Scatterplots of associations between prospectively ascertained adverse childhood experiences (ACEs) and **(A)** total surface area and **(B)** average cortical thickness. Scatterplots of associations between retrospectively reported ACEs and **(C)** total surface area and **(D)** average cortical thickness. Forest plots of standardized effect sizes (β and 95% confidence intervals) for the associations between prospectively ascertained ACEs and retrospectively reported ACEs with **(E)** surface area and **(F)** average cortical thickness. Solid squares mark standardized effect sizes for the independent associations between prospectively ascertained and retrospectively reported ACEs and age-45 brain structure, whereas open squares mark standardized effect sizes for the associations between prospectively ascertained and retrospectively reported ACEs and age-45 brain structure when controlling for the other ACEs measure.

Both prospectively ascertained and retrospectively reported ACEs were significantly associated with smaller total surface area (prospective β = −.12 [95% CI −.17, −.07], *p* < .001; retrospective β = −.06 [95% CI −.11, −.00], *p* = .03) and thinner average cortex (prospective β = −.13 [95% CI −.20, −.06], *p* < .001; retrospective β = −.09 [95% CI −.15, −.02], *p* = .01) at age 45 (Figure 1A–D). When entered into the same model, prospectively ascertained ACEs, but not retrospectively reported ACEs, were significantly associated with both measures of global brain structure (Figure 1E, F).

To further illustrate the preferential mapping of prospectively ascertained ACEs onto global measures of brain structure in midlife, we examined these associations within 2 distinct subgroups of Dunedin Study members (Figure S3). Among Dunedin Study members who retrospectively reported no ACEs (*n* = 299), those who—according to our prospective records—had more documented ACEs had smaller total surface area (β = −.15 [95% CI −.24, −.06], *p* = .001) and thinner average cortex (β = −.13 [95% CI −.24, −.02], *p* = .02). In contrast, among Dunedin Study members whose prospective records did not document any ACEs (*n* = 360), the associations between retrospectively reported ACEs and total surface area (β = −.08 [95% CI −.16, .01], *p* = .07) and average cortical thickness (β = −.05 [95% CI −.15, .05], *p* = .36) were not significant.

**Figure 2:**
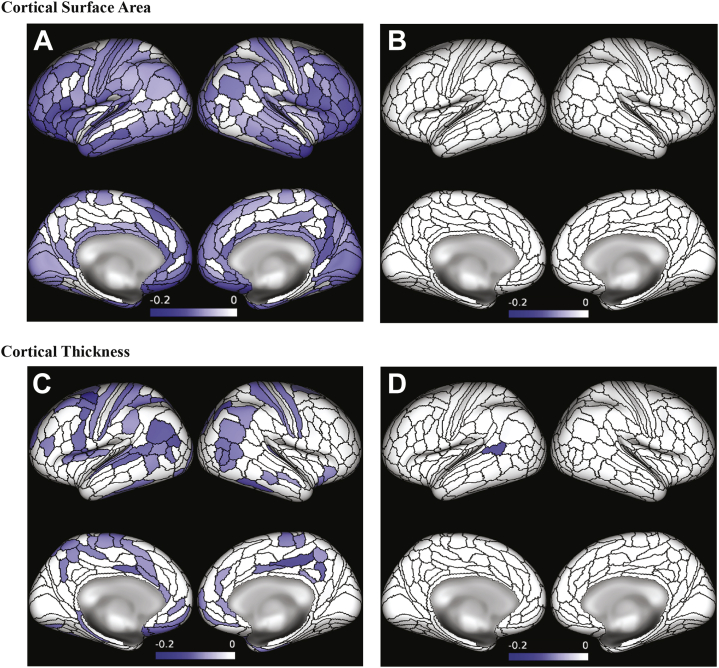
Associations between childhood adversity and parcelwise measures of cortical surface area and thickness. Parcelwise associations of **(A)** prospectively ascertained and **(B)** retrospectively reported adverse childhood experiences with surface area (*p* < .05, false discovery rate corrected). Parcelwise associations of **(C)** prospectively ascertained and **(D)** retrospectively reported adverse childhood experiences with cortical thickness (*p* < .05, false discovery rate corrected). Color bars reflect standardized effect sizes (β).

Given that ACEs were associated with decreased global measures of cortical surface area and thickness, we conducted a secondary set of analyses to explore the regional specificity of these associations. Prospectively ascertained ACEs were broadly associated with reduced cortical surface area; 357 of 360 parcels had negative effect sizes, and all 251 parcels surviving FDR correction showed a negative association between prospectively ascertained ACEs and surface area (Figure 2A). Consistent with the patterns observed for global measures, retrospectively reported ACEs were less strongly associated with reduced cortical surface area across the brain; although 332 parcels had negative effect sizes, none survived FDR correction (Figure 2B).

Prospectively ascertained ACEs were also broadly associated with reduced cortical thickness; 349 of 360 parcels had negative effect sizes, and all 85 parcels surviving FDR correction showed a negative association between prospectively ascertained ACEs and cortical thickness (Figure 2C). Again, retrospectively reported ACEs were less strongly associated with reduced cortical thickness across the brain; although 306 parcels had negative effect sizes, only one survived FDR correction (Figure 2D).

### Are Prospectively Ascertained and Retrospectively Reported ACEs Associated With Subcortical Gray Matter Volume?

**Figure 3:**
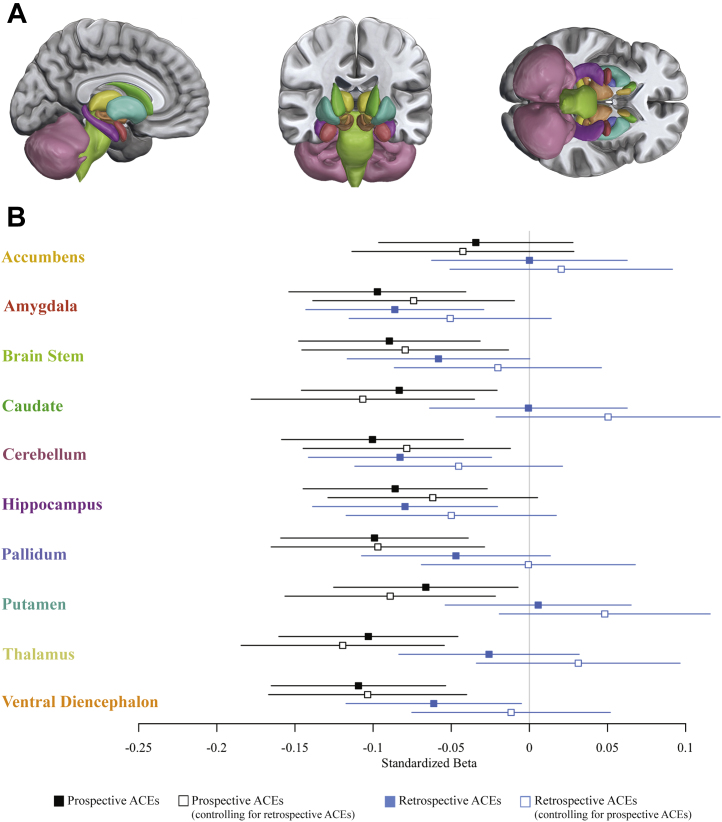
Associations between childhood adversity and subcortical gray matter volume. **(A)** The 10 subcortical structures for which gray matter volume was estimated. **(B)** Standardized effect sizes (β and 95% confidence intervals) for associations between prospectively ascertained adverse childhood experiences (ACEs) and retrospectively reported ACEs with average gray matter volume of 10 subcortical structures. Solid squares mark standardized effect sizes for the independent associations between prospectively ascertained and retrospectively reported ACEs and age-45 brain structure, whereas open squares mark standardized effect sizes for the associations between prospectively ascertained and retrospectively reported ACEs and age-45 brain structure when controlling for the other ACEs measure.

Prospectively ascertained ACEs were associated with smaller gray matter volume not only of a priori regions amygdala and hippocampus, but also of the brainstem, caudate, cerebellum, pallidum, putamen, thalamus, and ventral diencephalon. Retrospectively reported ACEs, however, were associated only with smaller gray matter volume of the amygdala, cerebellum, and hippocampus (Figure 3). When entered into the same model, prospectively ascertained ACEs, but not retrospectively reported ACEs, were significantly associated with smaller gray matter volume of the amygdala, brainstem, caudate, cerebellum, pallidum, putamen, thalamus, and ventral diencephalon (Figure 3; Figure S3).

### Are Associations Attributable to Perinatal Complications, Compromised Brain Health in Infancy, or Perceived Stress in Adulthood?

Prospectively ascertained ACEs continued to be significantly associated with total surface area and average cortical thickness, whereas retrospectively reported ACEs were not (Table S2). Prospectively ascertained ACEs also continued to be associated with smaller gray matter volume in 5 structures including the amygdala, cerebellum, pallidum, thalamus, and ventral diencephalon (with a trend toward an association with brainstem, caudate, and hippocampus), whereas retrospectively reported ACEs continued to be associated with smaller gray matter volume in only 2 structures, the amygdala and hippocampus (Table S3).

### Post Hoc Sensitivity Analyses

The following post hoc sensitivity analyses probed the robustness of the associations between prospectively ascertained ACEs and midlife brain structure revealed in primary analyses.

#### Does Total Intracranial Volume Influence Associations With Brain Structure?

Because our findings were nonspecific to particular brain areas, we expected that ACEs would no longer be statistically significantly associated with midlife brain structure if we controlled for intracranial volume. This was the case (Table S4).

#### Does Childhood SES Influence Associations With Brain Structure?

**Figure 4:**
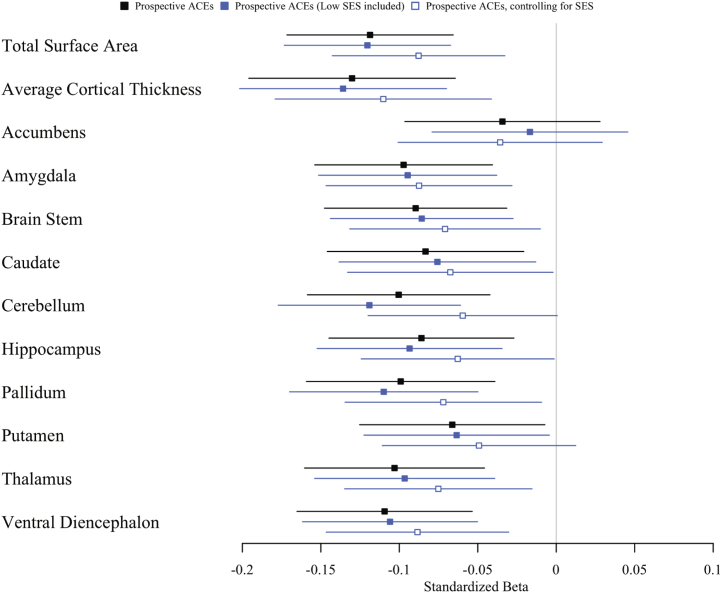
Influence of childhood socioeconomic status (SES) on associations between prospectively ascertained adversity and brain structure. Associations between prospectively ascertained adverse childhood experiences (ACEs) and age-45 brain structure are plotted with a black square, associations with SES included as ACEs are plotted with a filled blue square, and associations with SES included as a covariate are plotted with an open blue square. Forest plot shows standardized effect sizes (β and 95% confidence intervals).

We accounted for the fact that children with more ACEs tend to live in socioeconomically deprived circumstances. SES-adjusted point estimates of associations between ACEs and brain structure were similar to the nonadjusted estimates (Figure 4). After adjusting for SES, 7 of 11 associations remained significant, with the average effect size changing only from −.09 to −.07 (Table S5). The addition of socioeconomic deprivation as an extra ACE did not significantly increase associations with brain structure (Figure 4). The average effect size remained at −.09 (Table S5).

#### Do Different Adversities Contribute Disproportionately to Associations With Brain Structure?

**Figure 5:**
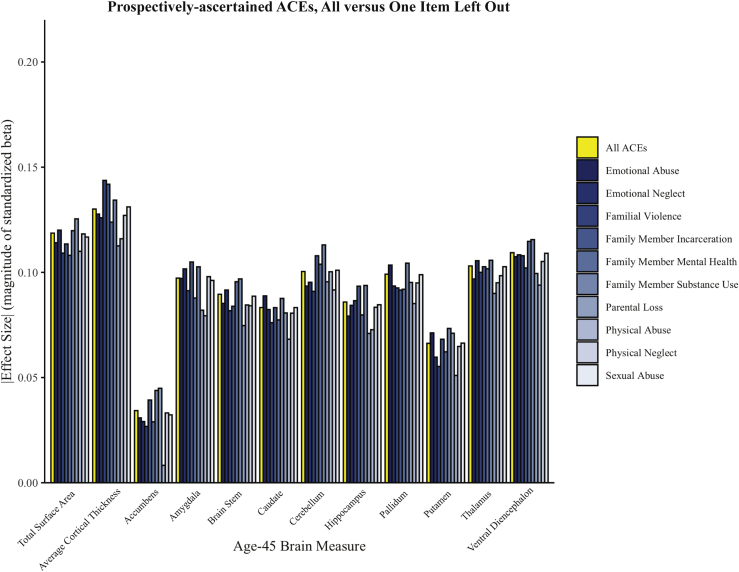
Associations between individual forms of adversity and brain structure. Prospectively ascertained adverse childhood experiences (ACEs) scores were recalculated excluding one item at a time. Standardized effect sizes for the associations between the newly calculated leave-one-out ACEs scores and each measure of age-45 brain structure are plotted in the bar graph. Associations with all ACEs are plotted in yellow, and associations with leave-one-out ACEs scores are plotted in shades of blue.

We conducted a leave-one-out analysis, in which we removed individual adversities in turn from the cumulative ACEs score. Removing any individual ACEs did not significantly alter the strength of associations with brain structure (Figure 5; Table S6).

#### Are Threat- and Deprivation-Specific Adversities Differentially Associated With Brain Structure?

**Figure 6:**
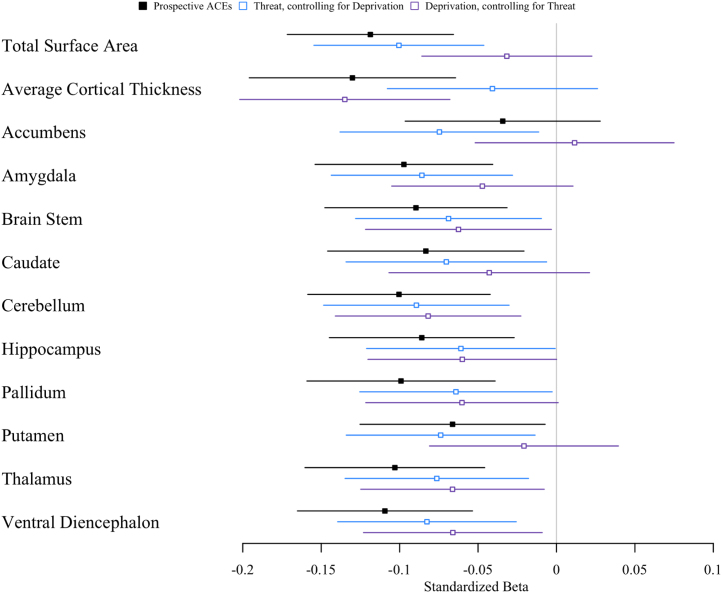
Associations between threat-specific and deprivation-specific adversity and brain structure. Associations between age-45 brain structure and all adverse childhood experiences (ACEs) are plotted with a black square; threat-specific ACEs, covarying for deprivation, are plotted with an open blue square; and deprivation-specific ACEs, covarying for threat, are plotted with an open purple square. Forest plot shows standardized effect sizes (β and 95% confidence intervals).

We tested a theoretical framework (56) positing that experiences of threat versus deprivation lead to different structural alterations (32). We did not observe consistent differences in the associations of threat- versus deprivation-specific adversities (Figure 6; Table S7). Total cortical surface area, average cortical thickness, and accumbens gray matter volume showed slightly different patterns of associations with threat- and deprivation-specific adversities, where threat-specific adversities were more strongly associated with smaller total cortical surface area and smaller accumbens volume, and deprivation-specific adversities were more strongly associated with thinner average cortex. For the remaining 9 structural measures, the point estimates for threat-specific adversities fell within the 95% CIs for the associations with deprivation-specific adversities and vice versa.

#### Are Associations Enriched Within Networks Along a Hierarchical Cortical Gradient?

**Figure 7:**
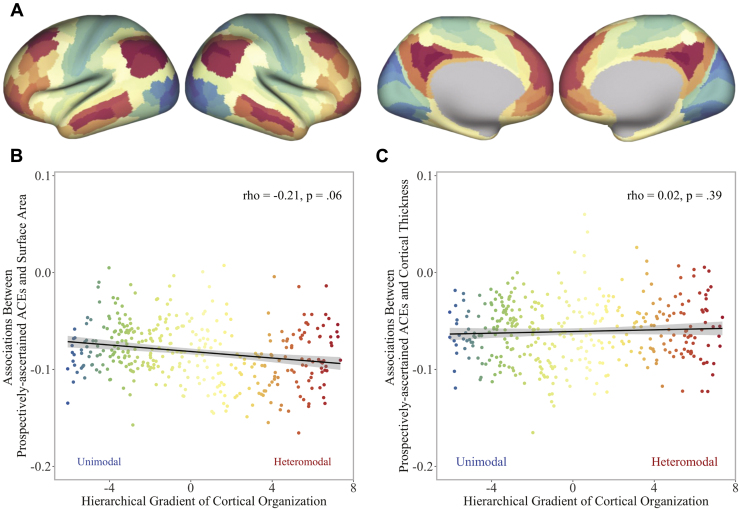
Correspondence of parcelwise associations with adversity along a cortical gradient of hierarchical information processing. **(A)** Cortical gradient capturing the macroscale hierarchical organization of information processing from basic sensory and somatomotor (cool colors) to higher cognitive (warm colors) functions (55). Standardized effect sizes for the associations between prospectively ascertained adverse childhood experiences (ACEs) and parcelwise **(B)** cortical surface area and **(C)** cortical thickness are plotted along the gradient.

Considering the widespread cortical differences identified in our primary analyses, we conducted a network enrichment analysis testing whether the parcelwise associations were enriched within specific networks along a cortical gradient of hierarchical information processing (Supplement; Figure 7A) (55). Although the strongest parcelwise associations between ACEs and surface area tended to cluster within heteromodal association areas, the association did not reach statistical significance (Spearman’s ρ = −.21, *p* = .06) (Figure 7B). The associations between ACEs and cortical thickness were likewise not enriched but were distributed evenly across unimodal and heteromodal areas (Spearman’s ρ = −.02, *p* = .39) (Figure 7C).

## Discussion

We analyzed data from 861 members of a birth cohort followed for 5 decades to test links between childhood adversity and midlife structural brain integrity. Our analyses revealed 5 main findings. First, both prospectively ascertained and retrospectively reported adversity were associated with smaller total cortical surface area and average cortical thickness as well as with smaller subcortical gray matter volume. Second, associations with prospectively ascertained adversity were consistently stronger than associations with retrospectively reported adversity. Third, associations with prospectively ascertained adversity remained significant even when controlling for retrospectively reported adversity, suggesting that adversity is associated with brain structure regardless of whether recalled or reported by adults. Fourth, parcelwise analyses revealed that associations between prospectively ascertained adversity and both surface area and thickness were not regionally specific, but rather widely distributed across cortex. Likewise, the associations between prospectively ascertained adversity and smaller subcortical gray matter volume were not localized and were apparent in 9 of the 10 structures examined.

Previous studies, including the largest study of childhood adversity to date (7), have demonstrated associations with smaller gray matter volume across a number of cortical regions (16,17,25, 26, 27). Our findings extend these prior associations by documenting that they encompass both surface area and cortical thickness. Our network enrichment analyses further extend prior findings by demonstrating that these associations are distributed relatively evenly across a cortical gradient of hierarchical information processing from basic sensory and somatomotor to higher cognitive functions. With regard to subcortical regions, adversity was associated with smaller gray matter volume not only of the amygdala and hippocampus—structures commonly targeted in prior studies of childhood adversity (15)—but also of the brainstem, caudate, cerebellum, pallidum, putamen, thalamus, and ventral diencephalon. Such widespread manifestations of adversity in the structural integrity of distributed cortical and subcortical brain regions supporting basic to complex affective, cognitive, sensory, and motoric processes are consistent with research demonstrating that adversity is associated with increased risk for all forms of mental disorders rather than any specific disorder (5).

The effect sizes we observed between childhood adversity and brain structure were small. However, this is one of the first studies to report effect sizes from a population-representative sample. More research using large, population-representative samples will be necessary to accurately estimate the magnitude of exposure-brain associations (57). Moreover, as we have recently shown, ACEs scores can forecast mean group differences in later health problems but have poor accuracy in identifying individuals at high risk for future health problems (58). However, small effect sizes may be consequential over the long term, either because effects accumulate over time or because many individuals are affected (59). Given the research design, the effect sizes reported herein are most likely reliable and, while small, may also be consequential.

Several sensitivity analyses were conducted to further probe the associations between childhood adversity and midlife brain structure. The observed associations between prospectively ascertained adversity and total cortical surface area, average cortical thickness, and half of the observed associations with subcortical gray matter volume were independent of potential preexisting developmental risks or low childhood SES that may influence brain development, as well as more proximal stressors near the time of neuroimaging. The observed associations were not driven by any particular type of adversity, but rather reflected relatively equal contributions of individual forms of adversity. Furthermore, threat- and deprivation-related adversities were largely associated with the same structural differences, with the exception of exposure-specific associations with global cortical measures and accumbens volume. Future research could investigate these differential associations.

This study is not without limitations. First, our observational study cannot establish causality or exclude all other potential explanations for the documented associations. For example, cortical thickness and surface area are under partial genetic influence (42), as are some forms of adversity (60), raising the possibility that common genes may unite the two. Second, we had only one wave of neuroimaging data, in midlife (61). Childhood neuroimaging could not be done in the Dunedin Study, which launched in 1972 before scanning was possible. Third, our findings are limited to a single, primarily white cohort born in the 1970s. Fourth, we were unable to investigate critical periods, as we did not know the exact timing of adversity. Fifth, it is possible that prospectively ascertained adversity was more strongly associated with structural brain integrity than retrospectively reported adversity because prospective measurement may detect relatively severe forms of adversity. However, prospective rates were lower than retrospective rates for the most severe adversity types, such as sexual abuse (Figure S2C). Sixth, following common practice in research and clinical settings, we summarized childhood adversity in a single index that did not distinguish between different adversities, which, in theory, may have different sequelae. However, recent analyses suggest considerable overlap across most types of adversity (62), and our post hoc sensitivity analyses indicated that this overlap extends to the associations of different types of adversity with brain structure. Seventh, it has previously been demonstrated that retrospective reports of adversity are more strongly related to self-reports of mental health, whereas prospectively ascertained measures of adversity are more strongly related to objective biomarkers of physical health (38). Our finding that prospective measures of adversity are more strongly related to objective measures of structural brain integrity, taken together with the fact that associations between adversity and mental health are stronger than those between adversity and brain structure (38,40), suggests that the link between adversity and mental health is not readily accounted for by brain structure as assessed in the current study.

These limitations notwithstanding, our findings have implications for theory, methodology, and policy. With respect to theory, the finding that childhood adversity is associated with structural differences in midlife brain is consistent with the hypothesis that early life stress may become biologically embedded (8, 9, 10, 11, 12, 13, 14). However, whereas initial research on the effects of chronic stress and adversity and the developing brain focused primarily on the hippocampus and later on other regions such as the amygdala and prefrontal cortex, we found that adversity-related structural differences are evident across the entire brain, suggesting that if adversity does become biologically embedded in the brain, it does not do so with much local specificity.

The widespread, nonlocalized nature of associations between childhood adversity and midlife structural brain integrity has implications for future studies of mechanisms through which adversity becomes biologically embedded. Specifically, it suggests focusing on broad developmental pathways and pathophysiological mechanisms that may be sensitive to childhood adversity. For example, disruptions to synaptogenesis, such as accelerated pruning, may result in long-term and widespread differences in structural brain integrity (9). Chronic stress may also impact global brain structure through sympathetic activation of a proinflammatory immune response (12). Such chronic inflammation may alter the activity of specialized cells and molecules that are part of the brain’s immune system, such as microglia, which are responsible for neuronal repair and clearing cellular debris (12,13). Microglia are also involved in neurodevelopmental processes such as synaptogenesis, neurogenesis, and synaptic pruning (13). Thus, chronic inflammation, particularly during critical neurodevelopmental periods, may be a pathway through which childhood adversity results in long-term, widespread, and nonlocalized brain alterations. Yet another possible pathway through which early environmental stress may alter the brain in a global manner is trauma-induced engagement in health-harming behaviors, such as smoking, heavy drinking, and poor nutrition. These health-harming behaviors have been demonstrated to have physiological effects (3), which may, in turn, broadly impact brain structure. Given these widespread differences in structural brain integrity, future studies should not restrict their analyses to a priori regions of interest. To be appropriately powered to conduct whole-brain analyses, future studies—both prospective and retrospective—will need to be larger. Currently, many studies examining the brain correlates of childhood adversity in children and adolescents include fewer than 100 participants (32), thereby limiting the power with which these studies can examine multiple structural features of the brain. Retrospective studies of adults may need even more participants, given that retrospective reporting underestimates associations between childhood adversity and midlife brain structure. Regardless, continuing to limit the scope of analyses to a few select regions of interest may paint a misleadingly localized picture of how childhood adversity becomes biologically embedded in the structure of the brain.

The findings of this study also have methodological implications. To date, studies investigating biological embedding of stress have mostly relied on retrospective reports of adversity [for a review, see (14)]. However, retrospectively reported measures of adversity do not correlate very highly with prospectively ascertained measures (34). It is generally thought that retrospective reports may inflate associations between childhood adversity and adult health because people with mental health problems may exaggerate, or be biased to remember, bad experiences (36,37). While that may be the case in relation to the link between childhood adversity and interview-assessed mental disorders, evidence suggests that prospectively ascertained adversity is more highly correlated with biomarker-assessed health than are retrospectively reported measures (38). Our findings extend this pattern to brain structure. Prospectively ascertained adversity was associated with midlife brain structure even in Dunedin Study members who did not report adversity retrospectively. This raises the possibility that previous studies relying on retrospective reports have underestimated the magnitude of associations between childhood adversity and brain structure. Future neuroimaging studies of pediatric populations should prospectively ascertain childhood adversity.

Lastly, our findings have implications for policy. Given consistent evidence linking childhood adversity to an increased health burden across the life span (1, 2, 3,63), there has been a rise in public health initiatives focused on screening in an effort to prevent health problems later in life (64). Although our findings do not directly speak to the importance of screening, they do suggest that childhood adversity is associated with persistent differences in biology detectable decades later and that retrospective screening reports underestimate the strength of this association. As life span continues to increase around the world, a better understanding of the long-term impact of childhood adversity on midlife brain structure may help ensure that health span increases as well.

## Supplementary Material

Key Resources Table

## References

[1] Felitti V.J., Anda R.F., Nordenberg D., Williamson D.F., Spitz A.M., Edwards V. (1998). Relationship of childhood abuse and household dysfunction to many of the leading causes of death in adults. The Adverse Childhood Experiences (ACE) Study. Am J Prev Med.

[2] Campbell J.A., Walker R.J., Egede L.E. (2016). Associations between adverse childhood experiences, high-risk behaviors, and morbidity in adulthood. Am J Prev Med.

[3] Hughes K., Bellis M.A., Hardcastle K.A., Sethi D., Butchart A., Mikton C. (2017). The effect of multiple adverse childhood experiences on health: A systematic review and meta-analysis. Lancet Public Health.

[4] Green J.G., McLaughlin K.A., Berglund P.A., Gruber M.J., Sampson N.A., Zaslavsky A.M., Kessler R.C. (2010). Childhood adversities and adult psychiatric disorders in the national comorbidity survey replication I: Associations with first onset of DSM-IV disorders. Arch Gen Psychiatry.

[5] Schaefer J.D., Moffitt T.E., Arseneault L., Danese A., Fisher H.L., Houts R. (2018). Adolescent victimization and early-adult psychopathology: Approaching causal inference using a longitudinal twin study to rule out noncausal explanations. Clin Psychol Sci.

[6] Wilson R.S., Arnold S.E. (2006). Childhood adversity and psychosocial adjustment in old age. Am J Geriatr Psychiatry.

[7] Gur R.E., Moore T.M., Rosen A.F.G., Barzilay R., Roalf D.R., Calkins M.E. (2019). Burden of environmental adversity associated with psychopathology, maturation, and brain behavior parameters in youths. JAMA Psychiatry.

[8] Fox S.E., Levitt P., Nelson C.A. (2010). How the timing and quality of early experiences influence the development of brain architecture. Child Dev.

[9] Nelson C.A., Gabard-Durnam L.J. (2020). Early adversity and critical periods: Neurodevelopmental consequences of violating the expectable environment. Trends Neurosci.

[10] McEwen B.S. (2012). Brain on stress: How the social environment gets under the skin. Proc Natl Acad Sci U S A.

[11] Frodl T., O’Keane V. (2013). How does the brain deal with cumulative stress? A review with focus on developmental stress, HPA axis function and hippocampal structure in humans. Neurobiol Dis.

[12] Danese A., McEwen B.S. (2012). Adverse childhood experiences, allostasis, allostatic load, and age-related disease. Physiol Behav.

[13] Danese A., Baldwin J.R. (2017). Hidden wounds? Inflammatory links between childhood trauma and psychopathology. Annu Rev Psychol.

[14] Teicher M.H., Samson J.A., Anderson C.M., Ohashi K. (2016). The effects of childhood maltreatment on brain structure, function and connectivity. Nat Rev Neurosci.

[15] Paquola C., Bennett M.R., Lagopoulos J. (2016). Understanding heterogeneity in grey matter research of adults with childhood maltreatment—a meta-analysis and review. Neurosci Biobehav Rev.

[16] Popovic D., Anne R., Dwyer D.B., Antonucci L.A., Eder J., Sanfelici R. (2020). Traces of trauma: A multivariate pattern analysis of childhood trauma, brain structure and clinical phenotypes. Biol Psychiatry.

[17] Lim L., Radua J., Rubia K. (2014). Gray matter abnormalities in childhood maltreatment: A voxel-wise meta-analysis. Am J Psychiatry.

[18] Rao U., Chen L.A., Bidesi A.S., Shad M.U., Thomas M.A., Hammen C.L. (2010). Hippocampal changes associated with early-life adversity and vulnerability to depression. Biol Psychiatry.

[19] Edmiston E.E., Wang F., Mazure C.M., Guiney J., Sinha R., Mayes L.C., Blumberg H.P. (2011). Corticostriatal-limbic gray matter morphology in adolescents with self-reported exposure to childhood maltreatment. Arch Pediatr Adolesc Med.

[20] Hodel A.S., Hunt R.H., Cowell R.A., Van Den Heuvel S.E., Gunnar M.R., Thomas K.M. (2015). Duration of early adversity and structural brain development in post-institutionalized adolescents. Neuroimage.

[21] Tottenham N., Hare T.A., Quinn B.T., McCarry T.W., Nurse M., Gilhooly T. (2010). Prolonged institutional rearing is associated with atypically larger amygdala volume and difficulties in emotion regulation. Dev Sci.

[22] Luby J.L., Tillman R., Barch D.M. (2019). Association of timing of adverse childhood experiences and caregiver support with regionally specific brain development in adolescents. JAMA Netw Open.

[23] Lupien S.J., Parent S., Evans A.C., Tremblay R.E., Zelazo P.D., Corbo V. (2011). Larger amygdala but no change in hippocampal volume in 10-year-old children exposed to maternal depressive symptomatology since birth. Proc Natl Acad Sci U S A.

[24] Hanson J.L., Nacewicz B.M., Sutterer M.J., Cayo A.A., Schaefer S.M., Rudolph K.D. (2015). Behavioral problems after early life stress: Contributions of the hippocampus and amygdala. Biol Psychiatry.

[25] Kelly P.A., Viding E., Wallace G.L., Schaer M., De Brito S.A., Robustelli B., McCrory E.J. (2013). Cortical thickness, surface area, and gyrification abnormalities in children exposed to maltreatment: Neural markers of vulnerability?. Biol Psychiatry.

[26] Gold A.L., Sheridan M.A., Peverill M., Busso D.S., Lambert H.K., Alves S. (2016). Childhood abuse and reduced cortical thickness in brain regions involved in emotional processing. J Child Psychol Psychiatry.

[27] McLaughlin K.A., Sheridan M.A., Winter W., Fox N.A., Zeanah C.H., Nelson C.A. (2014). Widespread reductions in cortical thickness following severe early-life deprivation: A neurodevelopmental pathway to attention-deficit/hyperactivity disorder. Biol Psychiatry.

[28] Mackes N.K., Golm D., Sarkar S., Kumsta R., Rutter M., Fairchild G. (2020). Early childhood deprivation is associated with alterations in adult brain structure despite subsequent environmental enrichment. Proc Natl Acad Sci U S A.

[29] Sheridan M.A., Fox N.A., Zeanah C.H., McLaughlin K.A., Nelson C.A. (2012). Variation in neural development as a result of exposure to institutionalization early in childhood. Proc Natl Acad Sci U S A.

[30] Bauer P.M., Hanson J.L., Pierson R.K., Davidson R.J., Pollak S.D. (2009). Cerebellar volume and cognitive functioning in children who experienced early deprivation. Biol Psychiatry.

[31] Mehta M.A., Golembo N.I., Nosarti C., Colvert E., Mota A., Williams S.C.R. (2009). Amygdala, hippocampal and corpus callosum size following severe early institutional deprivation: The English and Romanian Adoptees Study Pilot. J Child Psychol Psychiatry.

[32] McLaughlin K.A., Weissman D., Bitrán D. (2019). Childhood adversity and neural development: A systematic review. Annu Rev Dev Psychol.

[33] Teicher M.H., Samson J.A. (2016). Annual research review: Enduring neurobiological effects of childhood abuse and neglect. J Child Psychol Psychiatry.

[34] Baldwin J.R., Reuben A., Newbury J.B., Danese A. (2019). Agreement between prospective and retrospective measures of childhood maltreatment: A systematic review and meta-analysis. JAMA Psychiatry.

[35] Widom C.S., Morris S. (1997). Accuracy of adult recollections of childhood victimization. Part 2: Childhood sexual abuse. Psychol Assess.

[36] Susser E., Widom C.S. (2012). Still searching for lost truths about the bitter sorrows of childhood. Schizophr Bull.

[37] Colman I., Kingsbury M., Garad Y., Zeng Y., Naicker K., Patten S. (2016). Consistency in adult reporting of adverse childhood experiences. Psychol Med.

[38] Reuben A., Moffitt T.E., Caspi A., Belsky D.W., Harrington H., Schroeder F. (2016). Lest we forget: Comparing retrospective and prospective assessments of adverse childhood experiences in the prediction of adult health. J Child Psychol Psychiatry.

[39] Newbury J.B., Arseneault L., Moffitt T.E., Caspi A., Danese A., Baldwin J.R., Fisher H.L. (2018). Measuring childhood maltreatment to predict early-adult psychopathology: Comparison of prospective informant-reports and retrospective self-reports. J Psychiatr Res.

[40] Danese A., Widom C.S. (2020). Objective and subjective experiences of child maltreatment and their relationships with psychopathology. Nat Hum Behav.

[41] Button K.S., Ioannidis J.P., Mokrysz C., Nosek B.A., Flint J., Robinson E.S., Munafò M.R. (2013). Power failure: Why small sample size undermines the reliability of neuroscience. Nat Rev Neurosci.

[42] Panizzon M.S., Fennema-Notestine C., Eyler L.T., Jernigan T.L., Prom-Wormley E., Neale M. (2009). Distinct genetic influences on cortical surface area and cortical thickness. Cereb Cortex.

[43] Poulton R., Moffitt T.E., Silva P.A. (2015). The Dunedin Multidisciplinary Health and Development Study: Overview of the first 40 years, with an eye to the future. Soc Psychiatry Psychiatr Epidemiol.

[44] Richmond-Rakerd L.S., D’Souza S., Andersen S.H., Hogan S., Houts R.M., Poulton R. (2020). Clustering of health, crime and social-welfare inequality in 4 million citizens from two nations. Nat Hum Behav.

[45] Shalev I., Caspi A., Ambler A., Belsky D.W., Chapple S., Cohen H.J. (2014). Perinatal complications and aging indicators by midlife. Pediatrics.

[46] Caspi A., Houts R.M., Belsky D.W., Harrington H., Hogan S., Ramrakha S. (2016). Childhood forecasting of a small segment of the population with large economic burden. Nat Hum Behav.

[47] Cohen S., Kamarck T., Mermelstein R. (1983). A global measure of perceived stress. J Health Soc Behav.

[48] Glasser M.F., Coalson T.S., Robinson E.C., Hacker C.D., Harwell J., Yacoub E. (2016). A multi-modal parcellation of human cerebral cortex. Nature.

[49] Elliott M.L., Knodt A.R., Ireland D., Morris M.L., Poulton R., Ramrakha S. (2020). What is the test-retest reliability of common task-functional MRI measures? New empirical evidence and a meta-analysis. Psychol Sci.

[50] Romer A.L., Elliott M.L., Knodt A.R., Sison M.L., Ireland D., Houts R. (2021). Pervasively thinner neocortex as a transdiagnostic feature of general psychopathology. Am J Psychiatry.

[51] Elliott M.L., Belsky D.W., Knodt A.R., Ireland D., Melzer T.R., Poulton R. (2019). Brain-age in midlife is associated with accelerated biological aging and cognitive decline in a longitudinal birth cohort [published online ahead of print December 10]. Mol Psychiatry.

[52] Carlisi C.O., Moffitt T.E., Knodt A.R., Harrington H., Ireland D., Melzer T.R. (2020). Associations between life-course-persistent antisocial behaviour and brain structure in a population-representative longitudinal birth cohort. Lancet Psychiatry.

[53] R Core Team R Foundation for Statistical Computing. Vienna, Austria. https://www.r-project.org/.

[54] Benjamini Y., Hochberg Y. (1995). Controlling the false discovery rate: A practical and powerful approach to multiple testing. J R Stat Soc Ser B Methodol.

[55] Margulies D.S., Ghosh S.S., Goulas A., Falkiewicz M., Huntenburg J.M., Langs G. (2016). Situating the default-mode network along a principal gradient of macroscale cortical organization. Proc Natl Acad Sci U S A.

[56] McLaughlin K.A., Sheridan M.A., Lambert H.K. (2014). Childhood adversity and neural development: Deprivation and threat as distinct dimensions of early experience. Neurosci Biobehav Rev.

[57] Falk E.B., Hyde L.W., Mitchell C., Faul J., Gonzalez R., Heitzeg M.M. (2013). What is a representative brain? Neuroscience meets population science. Proc Natl Acad Sci U S A.

[58] Baldwin J.R., Caspi A., Meehan A.J., Ambler A., Arseneault L., Fisher H.L. (2021). Population vs individual prediction of poor health from results of adverse childhood experiences screening. JAMA Pediatr.

[59] Funder D.C., Ozer D.J. (2019). Evaluating effect size in psychological research: Sense and nonsense. Adv Methods Pract Psychol Sci.

[60] Dalvie S., Maihofer A.X., Coleman J.R.I., Bradley B., Breen G., Brick L.A. (2020). Genomic influences on self-reported childhood maltreatment. Transl Psychiatry.

[61] Danese A. (2020). Annual Research Review: Rethinking childhood trauma—new research directions for measurement, study design and analytical strategies. J Child Psychol Psychiatry.

[62] Smith K.E., Pollak S.D. (2021). Rethinking concepts and categories for understanding the neurodevelopmental effects of childhood adversity. Perspect Psychol Sci.

[63] Wilson R.S., Boyle P.A., Levine S.R., Yu L., Anagnos S.E., Buchman A.S. (2012). Emotional neglect in childhood and cerebral infarction in older age. Neurology.

[64] Lacey R.E., Minnis H. (2020). Practitioner Review: Twenty years of research with adverse childhood experience scores—advantages, disadvantages and applications to practice. J Child Psychol Psychiatry.

