# Supplementary material for: Long-term Neural Embedding of Childhood Adversity in a Population-Representative Birth Cohort Followed for 5 Decades
Source: Biol Psychiatry. 2021 Aug 1;90(3):182–93. doi: 10.1016/j.biopsych.2021.02.971 (PMC8274314; doi:10.1016/j.biopsych.2021.02.971)
Supplement: Supplementary Material [file mmc2.pdf]

Long-Term Neural Embedding of Childhood Adversity in a Population-Representative Birth Cohort Followed for Five Decades

Supplemental Information

Contents

Supplemental Text. Extended Methods and References…..2
Supplemental Figure S1. Dunedin Study cohort attrition analysis...5
Supplemental Figure S2. Distribution of ACEs in the Dunedin Study cohort 6
Supplemental Figure S3. Forest plots depicting the effect sizes (standardized ß and 95% confidence intervals) of associations between ACEs and age-45 global surface area, cortical thickness, and subcortical grey matter volume for those individuals with no ACEs documented prospectively or no ACEs reported retrospectively............................7
Supplemental Table S1. Correlations between covariates, ACEs, and age-45 brain structure..............................8
Supplemental Table S2. Standardized regression coefficients (ß) and 95% confidence intervals for associations between prospectively-ascertained and retrospectively-reported ACEs and age-45 global brain structure after controlling for perinatal complications, age-3 brain health, and adult perceived stress..9
Supplemental Table S3. Standardized regression coefficients (ß) and 95% confidence intervals for associations between prospectively-ascertained and retrospectively-reported ACEs and age-45 subcortical volume after controlling for perinatal complications, age-3 brain health, and adult perceived stress..10
Supplemental Table S4. Standardized regression coefficients (ß) and 95% confidence intervals for associations between prospectively-ascertained ACEs and age-45 subcortical volume, controlling for total intracranial volume…11
Supplemental Table S5. Standardized regression coefficients (ß) and 95% confidence intervals for associations between prospectively-ascertained ACEs and age-45 brain structure, presented in the leftmost column, as compared to those adjusted for SES (light grey) and those including low SES as an ACEs (dark grey)..12
Supplemental Table S6. Standardized regression coefficients (ß) and 95% confidence intervals for associations between prospectively-ascertained ACEs and age-45 brain structure, excluding each prospectively-ascertained ACEs from the total prospectively-ascertained ACEs score in turn…..13
Supplemental Table S7. Standardized regression coefficients (ß) and 95% confidence intervals for associations between prospectively-ascertained threat, prospectively-ascertained deprivation, prospectively-ascertained total ACEs, and age-45 brain structure. White columns denote bivariate associations, whereas grey columns present results from multiple regression models in which both threat and deprivation were included in the model.............................14

Supplementary Methods

Study Design and Sample

Participants are members of the Dunedin Study, a longitudinal investigation of health and behavior in a population-representative birth cohort. Study members (N=1,037; 91% of eligible births; 52% male) were all individuals born between April 1972 and March 1973 in Dunedin, New Zealand (NZ), who were eligible based on residence in the province and participation in the first assessment at age 3.[1] The cohort represented the full range of socioeconomic status in the general population of NZ's South Island and, as adults, matches the NZ National Health and Nutrition Survey on key adult health indicators (e.g., body mass index, smoking, general practitioner visits) and same-age citizens in the NZ Census on educational attainment.[2] The cohort is primarily white (93%). Data were available at birth and assessments were carried out at ages 3, 5, 7, 9, 11, 13, 15, 18, 21, 26, 32, 38, and most recently (completed April 2019) 45 years, when 94% (N=938) of the 997 living Study members participated. The relevant ethics committees approved each phase of the Study and written informed consent was obtained from all Study members. At each assessment, Study members were brought to the research unit for interviews and examinations. Neuroimaging was carried out at age 45 years in 93% (N=875) of participating Study members, who represented the original cohort on key demographic variables (attrition analysis in **Supplemental Figure S1**). Of the 875 Study members from whom imaging data were collected, four were excluded due to major incidental findings or previous injuries (e.g., large tumors or extensive damage to the brain/skull), nine due to missing FLAIR or field map scans (see below for details), and one due to poor surface mapping, yielding 861 neuroimaging datasets for our analyses.

Exposure to Adverse Childhood Experiences

We assessed ten categories of childhood adversity introduced by the CDC-Kaiser Permanente Adverse Childhood Experiences Study[3]: five types of child harm (physical abuse, emotional abuse, physical neglect, emotional neglect, and sexual abuse) and five types of household dysfunction (incarceration of a family member, household substance abuse, household mental illness, loss of a parent, and household partner violence). As previously described,[4] ACEs in the Dunedin Study have been assessed both prospectively and retrospectively, as detailed next.

Prospectively-ascertained ACEs were determined for each Study member from records collected at ages 3, 5, 7, 9, 11, 13 and 15 years.[4] These records include social services visits, structured interviews with the Study member and their parents, observed interactions between Study members and parents, observations of child well-being made by research staff at the time of assessment, self-reports collected from parents regarding parental criminality, notes from home visits, and notes from teachers that asked about the wellbeing of Study members. Prospectively-ascertained ACEs were available for all 861 participants with usable neuroimaging data.

Retrospectively-reported ACEs were derived from structured interviews conducted with Dunedin Study members at age 38 years.[4] Memories of physical, sexual, and emotional abuse, and physical and emotional neglect during childhood were ascertained by the Childhood Trauma Questionnaire (CTQ).[5] Memories of the five ACEs relating to household dysfunction during childhood were ascertained by the Family History Screen[6] and interview questions regarding household partner violence and parental loss. Retrospectively-reported ACEs were missing for 7 of the 861 Study members with usable neuroimaging data. Thus, analyses using prospectively-ascertained ACEs were conducted on data from 861 and those using retrospectively-reported ACEs on data from 854 Study members.

The number of these ten categories of adversities experienced was summed to yield a cumulative ACEs score between 1 and 10. Consistent with the reporting conventions used in this area of research,[3] and due to the relatively few children in the population who experience a very high number of ACEs, the 10-point ACE scale was consolidated, and both prospectively-ascertained and retrospectively-reported ACEs were coded 0, 1, 2, 3, or 4+ for all analyses in the current study. The distribution of ACEs for Study members included in the current analyses resembled that of the CDC-Kaiser Permanente ACE study (**Supplemental Figure S2A**),[3] and did not differ between men and women ($\chi^2(4, N=861) = 1.76$, $p = .78$ for prospectively-ascertained ACEs, $\chi^2(4, N=854) = 5.73$, $p = .22$ for retrospectively-reported ACEs; **Supplemental Figure S2B**). Consistent with methodological evaluations of measurement differences between prospective and retrospective reports,[7] prospectively-ascertained and retrospectively-reported ACEs were only modestly correlated among Study members with imaging data ($r=.48$, $p<.001$), and precise agreement between the number of adverse experiences prospectively-ascertained and retrospectively-reported was fair (weighted Kappa=.31 [95% CI:.27,.36]).

Covariates
Perinatal complications and childhood neurocognitive health were added into our regression models to test whether ACEs were associated with midlife brain structure after developmental risks in the prenatal or infancy periods may have exerted effects. *Perinatal complications* were assessed from hospital records and coded as the sum of the number of prenatal, intrapartum, and neonatal complications experienced.[8] At age 3 years, a composite measure of *childhood neurocognitive health* was derived from a 45-minute examination that included assessments by a pediatric neurologist, standardized tests of cognitive function, receptive language, motor skills, and examiners' ratings of emotional and behavioral regulation. Scores across these five domains were combined to create an age-3 Brain Health score.[9] Additionally, age-45 perceived stress was added into our models to test whether brain structure was associated with adverse events in childhood while discounting any potential effects of current stress. *Perceived stress* was assessed with the Perceived Stress Scale,[10] measuring the extent to which Study members felt stressed, unable to cope, and as if events occurring to them were uncontrollable and unexpected. These covariates were consistently and significantly associated with prospectively-ascertained and retrospectively-reported ACEs as well as with measures of midlife brain structure (**Supplemental Table S1**).

MRI Acquisition
Study members were scanned using a MAGNETOM Skyra 3T scanner (Siemens Healthcare GmbH) equipped with a 64-channel head/neck coil at the Pacific Radiology Group imaging center in Dunedin, New Zealand. High resolution T1-weighted images were obtained using an MP-RAGE sequence with the following parameters: TR = 2400 ms; TE = 1.98 ms; 208 sagittal slices; flip angle, 9°; FOV, 224 mm; matrix =256×256; slice thickness = 0.9 mm with no gap (voxel size 0.9×0.875×0.875 mm); and total scan time = 6 minutes and 52 seconds. 3D fluid-attenuated inversion recovery (FLAIR) images were obtained with the following parameters: TR = 8000 ms; TE = 399 ms; 160 sagittal slices; FOV = 240 mm; matrix = 232×256; slice thickness = 1.2 mm (voxel size 0.9×0.9×1.2 mm); and total scan time = 5 minutes and 38 seconds. Additionally, a gradient echo field map was acquired with the following parameters: TR = 712 ms; TE = 4.92 and 7.38 ms; 72 axial slices; FOV = 200 mm; matrix = 100×100; slice thickness = 2.0 mm (voxel size 2 mm isotropic); and total scan time = 2 minutes and 25 seconds.

Image Processing
The above structural MRI data were analyzed using the Human Connectome Project (HCP) minimal preprocessing pipeline as detailed elsewhere.[11] T1-weighted and FLAIR images were processed through the PreFreeSurfer, FreeSurfer, and PostFreeSurfer pipelines. T1-weighted and FLAIR images were corrected for readout distortion using the gradient echo field map, co-registered, brain-extracted, and aligned together in the native T1-space using boundary-based registration.[12] Images were then processed with a custom FreeSurfer recon-all pipeline optimized for structural MRI with higher resolution than 1 mm isotropic. Finally, recon-all output were converted into CIFTI format and registered to common 32k_FS_LR mesh using MSM-sulc.[13] Outputs of the minimal preprocessing pipeline were visually checked for accurate surface generation by examining each Study member's myelin map, pial surface, and white matter boundaries.

Statistical Analyses
Post Hoc Sensitivity Analyses
We conducted several post hoc sensitivity analyses to further probe the robustness of associations between prospectively-ascertained ACEs and midlife brain structure revealed in our primary analyses. First, we investigated associations while covarying for total intracranial volume (**Supplemental Table S4**). Second, we accounted for the fact that children with more ACEs were more likely to be raised in socioeconomically deprived circumstances by including childhood socioeconomic status (SES), measured with a six-point scale assessing parents' occupational status, categorized based upon the educational levels and income associated with that occupation in data from the New Zealand census,[14] as a covariate. We further modeled childhood SES as an additional form of adversity. For this analysis, Study members with scores on the lower third of the scale were classified as having experienced low SES and their prospectively-ascertained ACEs scores were increased by 1. Results from both sets of analyses are presented in **Figure 4** and **Supplemental Table S5**. Third, we examined whether the magnitude of associations differed based on the type of adversity experienced. We first conducted a leave-one-out analysis, in which we removed individual items from the ACEs score (**Figure 5**). Next, based on an emerging theoretical model[15] positing differences between experiences of threat and deprivation that give rise to adversity-specific structural alterations within circuits such as the fronto-amygdala, salience, and frontoparietal networks,[16] we examined separately associations with items representing threat (sum of the physical abuse, emotional abuse, sexual abuse, and witnessing domestic violence ACE items) or deprivation (sum of the physical neglect, emotional neglect, and parental loss ACE items). We conducted

both bivariate (in which we investigated the associations between threat- and deprivation-related ACEs and age-45 brain structure in separate models, each accounting for sex) and multivariate analyses (in which we investigated the independent associations between threat- and deprivation-related ACEs and age-45 brain structure in the same model, accounting for sex). The results of these analyses are presented in **Supplemental Table S7**. The domain-specific associations from the multivariate model are further reported in the main text (**Figure 6**).

Network Enrichment Analyses

We conducted network enrichment analyses in which we tested whether the parcel-wise associations between prospectively-ascertained ACEs scores and surface-based cortical measures enriched within specific networks along a cortical gradient of hierarchical information processing from basic sensory and somatomotor to higher cognitive functions.[17] As previously described,[18] we tested for correspondence between the two maps by first parcellating the connectivity gradient into the 360 HCP-MMP1.0 by taking the mean of each parcel. This parcellated gradient was then correlated with the parcel-wise maps of standardized betas for the associations between prospectively-ascertained ACEs and both cortical surface area and thickness. To determine significance for each correlation, we compared this value to a null distribution generated by spin permutation testing,[19,20] in which each of the maps of standardized effect sizes for the associations between prospectively-ascertained ACEs and surface-based cortical measures were randomly spherically rotated 1000 times and correlated with a randomly rotated map of the gradient. Results were considered significant at $p < 0.05$.

Supplementary References

1. Poulton R, Moffitt TE, Silva PA (2015): The Dunedin Multidisciplinary Health and Development Study: overview of the first 40 years, with an eye to the future. *Soc Psychiatry Psychiatr Epidemiol* 50: 679–693.
2. Richmond-Rakerd LS, D’Souza S, Andersen SH, Hogan S, Houts RM, Poulton R, *et al.* (2020): Clustering of health, crime and social-welfare inequality in 4 million citizens from two nations. *Nat Hum Behav* 4: 255–264.
3. Felitti VJ, Anda RF, Nordenberg D, Williamson DF, Spitz AM, Edwards V, *et al.* (1998): Relationship of childhood abuse and household dysfunction to many of the leading causes of death in adults. The Adverse Childhood Experiences (ACE) Study. *Am J Prev Med* 14: 245–258.
4. Reuben A, Moffitt TE, Caspi A, Belsky DW, Harrington H, Schroeder F, *et al.* (2016): Lest we forget: comparing retrospective and prospective assessments of adverse childhood experiences in the prediction of adult health. *J Child Psychol Psychiatry* 57: 1103–1112.
5. Bernstein DP, Fink L. (1998): Childhood Trauma Questionnaire: a retrospective self-report. San Antonio, TX: The Psychological Corporation.
6. Milne BJ, Caspi A, Crump R, Poulton R, Rutter M, Sears MR, Moffitt TE (2009): The validity of the family history screen for assessing family history of mental disorders. *Am J Med Genet B Neuropsychiatr Genet* 150B: 41–49.
7. Baldwin JR, Reuben A, Newbury JB, Danese A (2019): Agreement Between Prospective and Retrospective Measures of Childhood Maltreatment: A Systematic Review and Meta-analysis. *JAMA Psychiatry* 76: 584–593.
8. Shalev I, Caspi A, Ambler A, Belsky DW, Chapple S, Cohen HJ, *et al.* (2014): Perinatal Complications and Aging Indicators by Midlife. *Pediatrics* 134: e1315–e1323.
9. Caspi A, Houts RM, Belsky DW, Harrington H, Hogan S, Ramrakha S, *et al.* (2016): Childhood forecasting of a small segment of the population with large economic burden. *Nat Hum Behav* 1: 0005
10. Cohen S, Kamarck T, Mermelstein R (1983): A global measure of perceived stress. *J Health Soc Behav* 24: 385–396.
11. Glasser MF, Sotiropoulos SN, Wilson JA, Coalson TS, Fischl B, Andersson JL, *et al.* (2013): The minimal preprocessing pipelines for the Human Connectome Project. *Neuroimage* 80: 105–124.
12. Greve DN, Fischl B (2009): Accurate and robust brain image alignment using boundary-based registration. *Neuroimage* 48: 63–72.
13. Robinson EC, Jbabdi S, Glasser MF, Andersson J, Burgess GC, Harms MP, *et al.* (2014): MSM: a new flexible framework for Multimodal Surface Matching. *Neuroimage* 100: 414–426.
14. Elley W, Irving J (1976). Revised socio-economic index for New Zealand. *N Z J Educ Stud* 11: 25-36.
15. McLaughlin KA, Sheridan MA (2016): Beyond Cumulative Risk: A Dimensional Approach to Childhood Adversity. *Curr Dir Psychol Sci* 25: 239–245.
16. McLaughlin KA, Weissman D, Bitrán D (2019): Childhood Adversity and Neural Development: A Systematic Review. *Annu Rev Dev Psychol* 1: 277–312.
17. Margulies DS, Ghosh SS, Goulas A, Falkiewicz M, Huntenburg JM, Langs G, *et al.* (2016): Situating the default-mode network along a principal gradient of macroscale cortical organization. *PNAS* 113: 12574–12579.
18. Romer AL, Elliott ML, Knodt AR, Sison ML, Ireland D, Houts R, *et al.* (2020): Pervasively Thinner Neocortex as a Transdiagnostic Feature of General Psychopathology. *AJP* appi.ajp.2020.19090934.
19. Alexander-Bloch AF, Shou H, Liu S, Satterthwaite TD, Glahn DC, Shinohara RT, *et al.* (2018): On testing for spatial correspondence between maps of human brain structure and function. *Neuroimage* 178: 540–551.
20. Váša F, Bullmore ET, Patel AX (2018): Probabilistic thresholding of functional connectomes: Application to schizophrenia. *Neuroimage* 172: 326–340.

Supplemental Figure S1. Dunedin Study cohort attrition analysis. We conducted an attrition analysis using childhood IQ, childhood SES, and Adverse Childhood Experiences (ACEs) to determine whether participants in the Phase 45 data collection were representative of the original cohort. A) No significant differences in childhood IQ were found between the full cohort, those still alive, those seen at Phase 45 or those scanned at Phase 45. Those who were deceased by the Phase 45 data collection had significantly lower childhood IQ's than those who were still alive (t = 2.09, $p = 0.04$). B) No significant differences were found between the full cohort, those deceased, those alive, those seen at Phase 45 or those scanned at Phase 45 on childhood SES. C) No significant differences were found between the full cohort, those deceased, those alive, those seen at Phase 45 or those scanned at Phase 45 on Adverse Childhood Events (ACEs).

A) **B)** **C)**

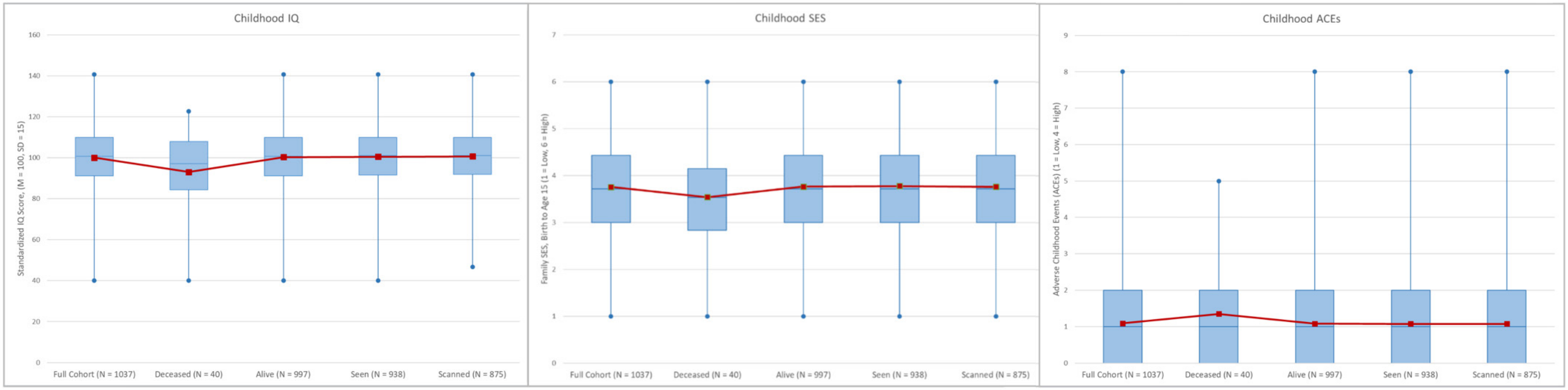

Supplemental Figure S2. Distribution of ACEs in the Dunedin Study cohort. Panel A) depicts the distribution of ACEs in the Dunedin cohort recorded prospectively and retrospectively, with comparison to ACEs distributions reported in the CDC ACEs Study. Distribution of ACEs in the CDC ACEs Study from Table 3 of Felitti et al. (1998, p. 251). Panel B) shows the number of men and women within each ACEs group for both prospectively-ascertained ACEs (left) and retrospectively-reported ACEs (right). There were no sex differences between the groups, $\chi^2(4, N=861) = 1.76$, $p = .78$, $\chi^2(4, N=854) = 5.73$, $p = .22$ for prospectively-ascertained and retrospectively-reported ACEs, respectively. Panel C) depicts the prevalence of individual ACEs recorded prospectively and retrospectively.

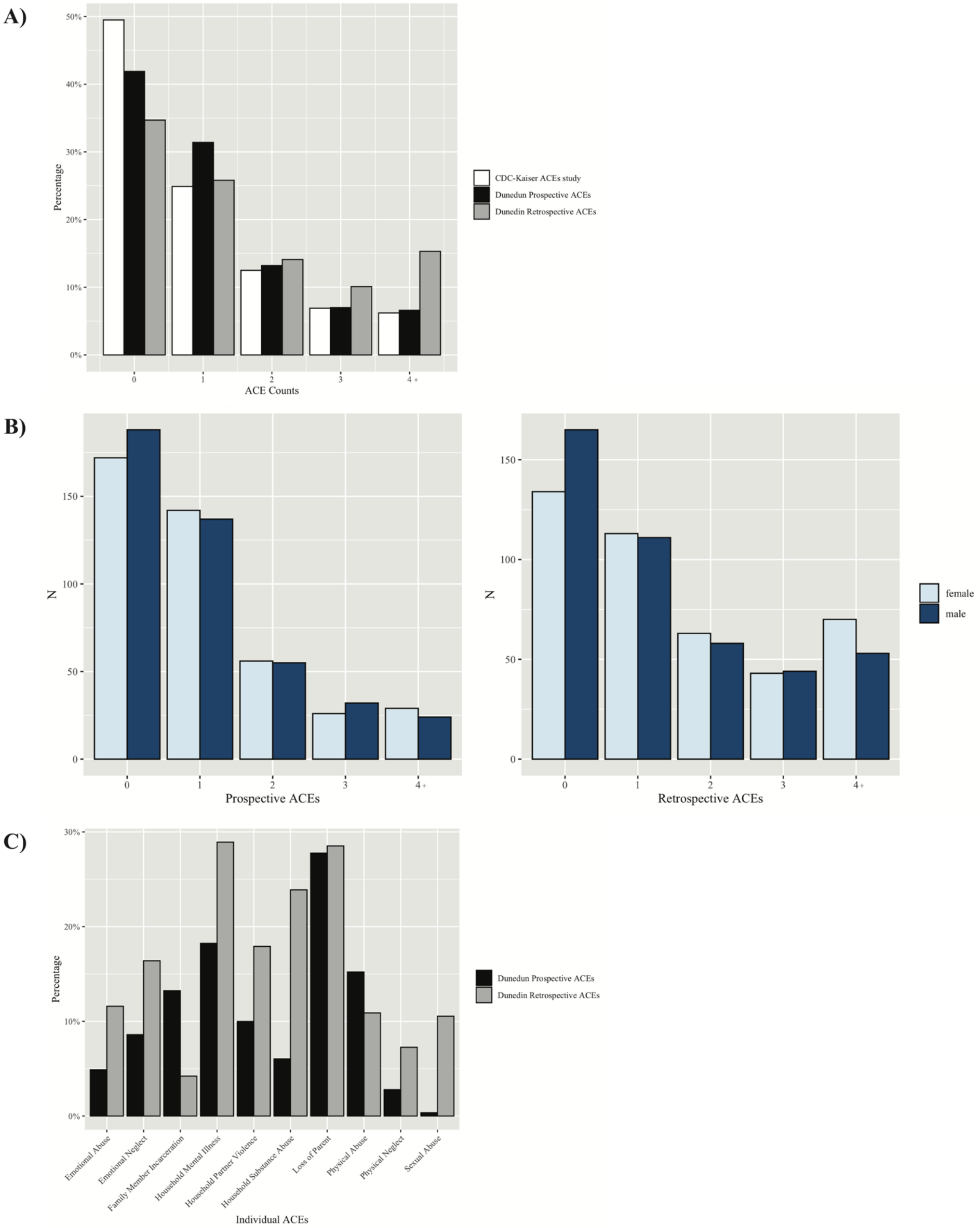

Supplemental Figure S3. Associations between number of prospectively-ascertained versus retrospectively-reported ACEs and brain structure. Brain structure outcome measures shown include A) total cortical surface area, B) average cortical thickness, and C) subcortical volume. The forest plots show the associations between prospectively-ascertained ACEs with age-45 brain structure for those individuals who reported no retrospectively-reported ACEs (N=299), in black, and the associations between retrospectively-reported ACEs with age-45 brain structure for those individuals who had no documented ACEs according to our prospectively-ascertained records (N=360), in blue. The forest plots show standardized regression coefficients (ß) and 95% confidence intervals. In panel C), associations significant at $p < .05$ after FDR-correction are drawn with a diamond.

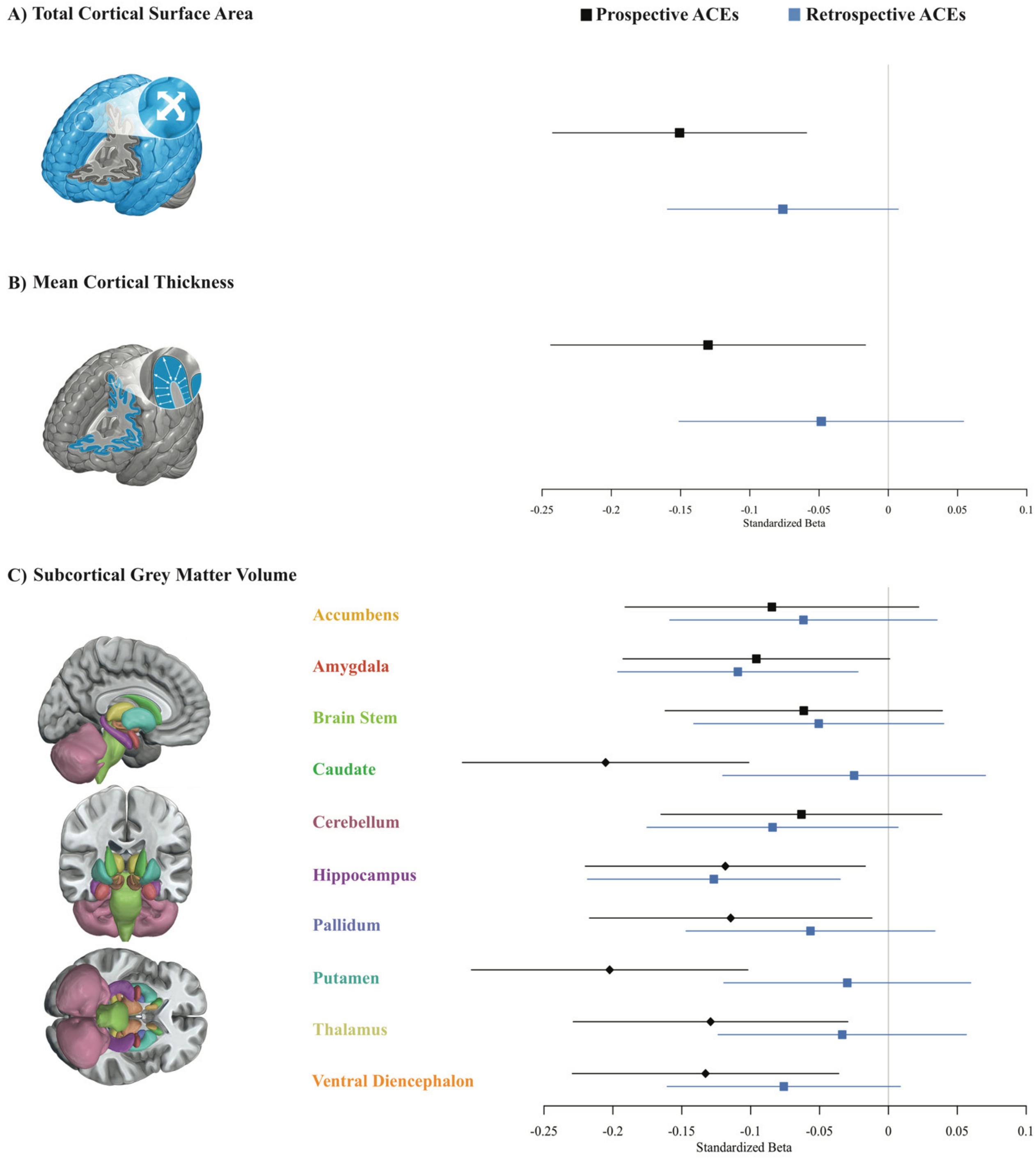

Supplemental Table S1. Correlations between covariates, ACEs, and age-45 global brain structure. Table depicting each covariate's relationship to adverse childhood experiences (prospectively-ascertained ACEs and retrospectively-reported ACEs) and midlife brain structure (total cortical surface area, mean cortical thickness, and subcortical grey matter volume). Pearson correlation coefficients, confidence intervals, and *p* values for the zero-order correlations are reported.

	**Birth – 15 Adverse Childhood Experiences**		**Age-45 Brain Outcome Measures**											
**Covariate**	**Prospective**	**Retro-spective**	**Total Surface Area**	**Mean Cortical Thickness**	**Accumbens GMV**	**Amygdala GMV**	**Brainstem GMV**	**Caudate GMV**	**Cerebellum GMV**	**Hippo-campus GMV**	**Pallidum GMV**	**Putamen GMV**	**Thalamus GMV**	**V. Dien-cephalon GMV**
**Perinatal Compli-cations**	r = 0.09<br>[0.02,0.16]<br>*p<.01*	r = 0.04<br>[-0.03,0.10]<br>*p=.29*	r = -0.11<br>[-0.18,-0.04]<br>*p<.01*	r = -0.02<br>[-0.08,0.05]<br>*p=.66*	r = -0.09<br>[-0.15,-0.02]<br>*p < .05*	r = -0.05<br>[-0.11,0.02]<br>*p = .18*	r = -0.16<br>[-0.22,-0.09]<br>*p < .001*	r = -0.11<br>[-0.18,-0.04]<br>*p<.01*	r = -0.10<br>[-0.17,-0.04]<br>*p<.01*	r = -0.09<br>[-0.15,-0.02]<br>*p < .05*	r = -0.11<br>[-0.17,-0.04]<br>*p<.01*	r = -0.10<br>[-0.17,-0.04]<br>*p<.01*	r = -0.12<br>[-0.19,-0.05]<br>*p<.001*	r = -0.15<br>[-0.21,-0.08]<br>*p < .001*
**Age-3 Brain Health**	r = -0.19<br>[-0.25,-0.13]<br>*p<.001*	r = -0.10<br>[-0.16,-0.03]<br>*p<.01*	r = 0.12<br>[0.05,0.18]<br>*p<.001*	r = 0.10<br>[0.03,0.16]<br>*p<.01*	r = 0.05<br>[-0.01,0.12]<br>*p = .13*	r = 0.08<br>[0.01,0.14]<br>*p < .05*	r = 0.14<br>[0.07,0.21]<br>*p < .001*	r = 0.10<br>[0.03,0.16]<br>*p<.01*	r = 0.11<br>[0.04,0.17]<br>*p<.01*	r = 0.13<br>[0.06,0.19]<br>*p<.001*	r = 0.12<br>[0.06,0.19]<br>*p<.001*	r = 0.08<br>[0.01,0.14]<br>*p < .05*	r = 0.15<br>[0.08,0.21]<br>*p < .001*	r = 0.13<br>[0.06,0.19]<br>*p<.001*
**Age-45 Perceived Stress**	r = 0.14<br>[0.07,0.20]<br>*p<.001*	r = 0.23<br>[0.17,0.30]<br>*p<.001*	r = -0.05<br>[-0.11 0.02]<br>*p=.16*	r = -0.14<br>[-0.21,-0.07]<br>*p<.001*	r = -0.03<br>[-0.10,0.04]<br>*p = .36*	r = -0.07<br>[-0.13,-0.00]<br>*p < .05*	r = -0.05<br>[-0.11,0.02]<br>*p=.16*	r < -0.00<br>[-0.07,0.06]<br>*p = .94*	r = -0.09<br>[-0.16,-0.03]<br>*p < .01*	r = -0.01<br>[-0.07,0.06]<br>*p = .86*	r = -0.01<br>[-0.08,0.05]<br>*p = .71*	r = -0.03<br>[-0.09,0.04]<br>*p = .46*	r = -0.07<br>[-0.14,-0.01]<br>*p < .05*	r = -0.05<br>[-0.12,0.02]<br>*p=.13*

Supplemental Table S2. Associations between ACEs and age-45 brain structure. The table shows results from multivariable regression models. Model I shows standardized regression coefficients (ß), 95% confidence intervals, and *p* values for prospectively-ascertained ACEs along with the covariates of perinatal complications, age-3 brain health, and adult perceived stress. Model II shows standardized regression coefficients (ß), 95% confidence intervals, and *p* values for retrospectively-reported ACEs along with the covariates of perinatal complications, age-3 brain health, and adult perceived stress.

		Age-45 Brain Structure Outcome Measures			
		**Total Cortical Surface Area**		**Mean Cortical Thickness**	
	**Predictor Variables**	β Estimate [95% Confidence Interval]	*p* value	β Estimate [95% Confidence Interval]	*p* value
**Model I**	**Prospective ACEs**	-0.09 [-0.15 - -0.04]	< .001	-0.10 [-0.17 - -0.04]	< .01
	Perinatal Complications	-0.05 [-0.11 - 0.00]	.05	0.02 [-0.05 - 0.08]	.63
	Age-3 Brain Health	0.13 [0.08 - 0.19]	< .001	0.07 [0.01 - 0.14]	< .05
	Perceived Stress	0.04 [-0.01 - 0.09]	.15	-0.11 [-0.18 - -0.04]	< .01
**Model II**	**Retrospective ACEs**	-0.05 [-0.10 - 0.00]	.07	-0.05 [-0.12 - 0.01]	.12
	Perinatal Complications	-0.05 [-0.11 - -0.00]	< .05	0.02 [-0.05 - 0.08]	.65
	Age-3 Brain Health	0.14 [0.09 - 0.19]	< .001	0.09 [0.02 - 0.16]	< .01
	Perceived Stress	0.04 [-0.01 - 0.09]	.18	-0.11 [-0.17 - -0.04]	< .01

Supplemental Table S3. Associations between ACEs and age-45 subcortical grey matter volume. The table shows results from multivariable regression models. Model I shows standardized regression coefficients (ß) and 95% confidence intervals for prospectively-ascertained ACEs along with the covariates of perinatal complications, age-3 brain health, and adult perceived stress. Model II shows standardized regression coefficients (ß) and 95% confidence intervals for retrospectively-reported ACEs along with the covariates of perinatal complications, age-3 brain health, and adult perceived stress. Associations significant at $p < .05$, $p < .01$, and $p < .001$ after FDR correction are marked with asterisks.

		**Age-45 Grey Matter Volume Measures of Subcortical Regions**									
		**Accumbens**	**Amygdala**	**Brain Stem**	**Caudate**	**Cerebellum**	**Hippo-campus**	**Pallidum**	**Putamen**	**Thalamus**	**Ventral Dien-cephalon**
	**Predictor Variables**	β Estimate [95% CI]	β Estimate [95% CI]	β Estimate [95% CI]	β Estimate [95% CI]	β Estimate [95% CI]	β Estimate [95% CI]	β Estimate [95% CI]	β Estimate [95% CI]	β Estimate [95% CI]	β Estimate [95% CI]
**Model I**	**Prospective ACEs**	-0.02 [-0.08, 0.05]	-0.08 [-0.14, -0.02]*	-0.06 [-0.11, 0.00]	-0.07 [-0.13, -0.00]	-0.07 [-0.13, -0.01]*	-0.06 [-0.12, -0.00]	-0.08 [-0.14, -0.02]*	-0.05 [-0.11, 0.01]	-0.07 [-0.13, -0.01]*	-0.08 [-0.14, -0.02]*
	Perinatal Complications	-0.05 [-0.11, 0.01]	0.01 [-0.05, 0.07]	-0.10 [-0.16, -0.04]**	-0.06 [-0.13, 0.00]	-0.05 [-0.11, 0.01]	-0.03 [-0.09, 0.03]	-0.04 [-0.10, 0.02]	-0.06 [-0.12, 0.00]	-0.06 [-0.12, -0.00]	-0.08 [-0.14, -0.03]*
	Age-3 Brain Health	0.06 [-0.00, 0.13]	0.09 [0.03, 0.15]**	0.14 [0.08, 0.20]***	0.10 [0.03, 0.16]**	0.11 [0.05, 0.17]***	0.14 [0.08, 0.20]***	0.13 [0.07, 0.19]***	0.09 [0.03, 0.15]**	0.15 [0.09, 0.21]***	0.12 [0.07, 0.18]***
	Perceived Stress	0.01 [-0.05, 0.08]	< 0.00 [-0.05, 0.06]	0.03 [-0.03, 0.08]	0.05 [-0.01, 0.12]	-0.03 [-0.09, 0.03]	0.07 [0.01, 0.13]	0.05 [-0.01, 0.11]	0.04 [-0.02, 0.10]	< 0.00 [-0.05, 0.06]	0.03 [-0.03, 0.08]
**Model II**	**Retrospective ACEs**	0.01 [-0.06, 0.07]	-0.08 [-0.14, -0.02]*	-0.05 [-0.11, 0.01]	< 0.00 [-0.06, 0.07]	-0.07 [-0.13, -0.01]	-0.08 [-0.14, -0.02]*	-0.04 [-0.11, 0.02]	0.01 [-0.05, 0.07]	< -0.00 [-0.06, 0.05]	-0.05 [-0.11, 0.01]
	Perinatal Complications	-0.05 [-0.12, 0.01]	< 0.00 [-0.05, 0.06]	-0.11 [-0.16, -0.05]**	-0.07 [-0.13, -0.00]	-0.05 [-0.11, 0.01]	-0.04 [-0.09, 0.02]	-0.05 [-0.11, 0.01]	-0.06 [-0.12, 0.00]	-0.06 [-0.12, -0.01]	-0.09 [-0.14, -0.03]**
	Age-3 Brain Health	0.07 [0.00, 0.13]*	0.10 [0.04, 0.16]**	0.14 [0.09, 0.20]***	0.11 [0.04, 0.17]**	0.11 [0.05, 0.17]***	0.15 [0.09, 0.21]***	0.14 [0.08, 0.20]***	0.10 [0.04, 0.16]**	0.16 [0.11, 0.22]***	0.13 [0.08, 0.19]***
	Perceived Stress	0.01 [-0.06, 0.07]	0.01 [-0.05, 0.07]	0.03 [-0.03, 0.09]	0.04 [-0.02, 0.11]	-0.02 [-0.08, 0.04]	0.08 [0.02, 0.14]	0.05 [-0.01, 0.11]	0.03 [-0.03, 0.09]	< -0.00 [-0.06, 0.06]	0.03 [-0.03, 0.09]

Supplemental Table S4. Associations between ACEs and age-45 subcortical volume controlling for total ICV. Table of standardized regression coefficients (ß) and 95% confidence intervals for associations between prospectively-ascertained ACEs and age-45 subcortical volume, after controlling for total ICV. Associations significant at $p < .05$, $p < .01$, and $p < .001$ after FDR correction are marked with asterisks.

Subcortical ROIs	**Prospective ACEs (controlling for sex)**	**Prospective ACEs (controlling for sex + ICV)**
	β Estimate (95% CI)	β Estimate (95% CI)
Accumbens	-.03 (-.10, .03)	.01 (-.04, .07)
Amygdala	-.10** (-.15, -.04)	-.04 (-.09, .01)
Brain Stem	-.09** (-.15, -.03)	-.02 (-.07, .02)
Caudate	-.08* (-.15, -.02)	-.02 (-.07, .03)
Cerebellum	-.10** (-.16, -.04)	-.05 (-.11, .00)
Hippocampus	-.09** (-.14, -.03)	-.03 (-.08, .02)
Pallidum	-.10** (-.16, -.04)	-.03 (-.08, .02)
Putamen	-.07* (-.13, -.01)	-.01(-.06, .04)
Thalamus	-.10** (-.16, -.05)	-.03 (-.07, .01)
Ventral Diencephalon	-.11** (-.17, -.05)	-.04 (-.08, .00)

Supplemental Table S5. Associations between number of prospectively-ascertained ACEs and age-45 brain structure accounting for SES. Table of standardized regression coefficients (ß) and 95% confidence intervals for associations between prospectively-ascertained ACEs and age-45 brain measures, presented in the leftmost column, as compared to those adjusted for SES (light grey) and those including low SES as an ACEs (dark grey). Associations significant at p < .05, p < .01, and p < .001 after FDR correction are marked with asterisks.

		Prospective Measures		
		**Unadjusted (Prospective ACEs + sex)**	**Adjusted for SES**	**Prospective ACEs (including low SES + sex)**
**Age-45 Brain Structure**	Total Cortical Surface Area	-.12*** (-.17, -.07)	-.09* (-.14, -.03)	-.12*** (-.17, -.07)
	Average Cortical Thickness	-.13*** (-.20, -.06)	-.11* (-.18, -.04)	-.14*** (-.20, -.07)
	Accumbens Volume	-.03 (-.10, .03)	-.04 (-.10, .03)	-.02 (-.08, .05)
	Amygdala Volume	-.10** (-.15, -.04)	-.09* (-.15, -.02)	-.09** (-.15, -.04)
	Brain Stem Volume	-.09** (-.15, -.03)	-.07* (-.13, -.01)	-.09** (-.14, -.03)
	Caudate Volume	-.08* (-.15, -.02)	-.07 (-.13, -.00)	-.08* (-.14, -.01)
	Cerebellum Volume	-.10** (-.16, -.04)	-.06 (-.12, .00)	-.12*** (-.18, -.06)
	Hippocampus Volume	-.09** (-.14, -.03)	-.06 (-.12, -.00)	-.09** (-.15, -.03)
	Pallidum Volume	-.10** (-.16, -.04)	-.07* (-.13, -.01)	-.11** (-.17, -.05)
	Putamen Volume	-.07* (-.13, -.01)	-.05 (-.11, .01)	-.06* (-.12, -.00)
	Thalamus Volume	-.10** (-.16, -.05)	-.07* (-.13, -.02)	-.10** (-.15, -.04)
	Ventral Diencephalon Volume	-.11*** (-.17, -.05)	-.09* (-.15, -.03)	-.11** (-.16, -.05)

Supplemental Table S6. Associations between number of prospectively-ascertained ACEs and age-45 brain structure excluding each prospectively-ascertained ACEs from the total ACEs score in turn. Standardized regression coefficients (ß) and 95% confidence intervals are reported. Associations significant at $p < .05$, $p < .01$, and $p < .001$ after FDR correction are marked with asterisks.

	Total Cortical Surface Area	Average Cortical Thickness	Accumbens	Amygdala	Brain Stem	Caudate	Cere-bellum	Hippo-campus	Pallidum	Putamen	Thalamus	Ventral Dien-cephalon
**Predictor Variables**	***β* Estimate [95% CI]**	***β* Estimate [95% CI]**	***β* Estimate [95% CI]**	***β* Estimate [95% CI]**	***β* Estimate [95% CI]**	***β* Estimate [95% CI]**	***β* Estimate [95% CI]**	***β* Estimate [95% CI]**	***β* Estimate [95% CI]**	***β* Estimate [95% CI]**	***β* Estimate [95% CI]**	***β* Estimate [95% CI]**
**Prospective ACEs**	**-.12*** [-.17, -.07]**	**-.13*** [-.20, -.06]**	**-.03 [-.10, .03]**	**-.10** [-.15, -.04]**	**-.09** [-.15, -.03]**	**-.08* [-.15, -.02]**	**-.10** [-.16, -.04]**	**-.09** [-.14, -.03]**	**-.10** [-.16, -.04]**	**-.07* [-.13, -.01]**	**-.10** [-.16, -.05]**	**-.11*** [-.17, -.05]**
**ProACEs,** **excluding:** Physical Abuse	-.11*** [-.16, -.06]	-.12** [-.18, -.05]	-.01 [-.07, .05]	-.08** [-.14, -.02]	-.08** [-.14, -.03]	-.07* [-.13, -.01]	-.10** [-.16, -.04]	-.07* [-.13, -.01]	-.09** [-.15, -.03]	-.05 [-.11, .01]	-.10** [-.15, -.04]	-.09** [-.15, -.04]
Physical Neglect	-.12*** [-.17, -.07]	-.13*** [-.19, -.06]	-.03 [-.10, .03]	-.10** [-.15, -.04]	-.08** [-.14, -.03]	-.08* [-.14, -.02]	-.09** [-.15, -.03]	-.08** [-.14, -.02]	-.09** [-.16, -.03]	-.06* [-.12, -.01]	-.10** [-.16, -.04]	-.11*** [-.16, -.05]
Emotional Abuse	-.11*** [-.17, -.06]	-.13*** [-.19, -.06]	-.03 [-.09, .03]	-.10** [-.15, -.04]	-.09** [-.14, -.03]	-.09** [-.15, -.03]	-.09** [-.15, -.04]	-.08* [-.14, -.02]	-.10** [-.16, -.04]	-.07* [-.13, -.01]	-.10** [-.15, -.04]	-.11*** [-.16, -.05]
Emotional Neglect	-.12*** [-.17, -.07]	-.13*** [-.19, -.06]	-.03 [-.09, .03]	-.10** [-.16, -.05]	-.09** [-.15, -.03]	-.08* [-.14, -.02]	-.10** [-.15, -.04]	-.08** [-.14, -.03]	-.09** [-.15, -.03]	-.06 [-.12, -.00]	-.11*** [-.16, -.05]	-.11*** [-.16, -.05]
Sexual Abuse	-.12*** [-.17, -.06]	-.13*** [-.20, -.07]	-.03 [-.09, .03]	-.10** [-.15, -.04]	-.09** [-.15, -.03]	-.08* [-.15, -.02]	-.10** [-.16, -.04]	-.08** [-.14, -.03]	-.10** [-.16, -.04]	-.07* [-.13, -.01]	-.10** [-.16, -.05]	-.11*** [-.16, -.05]
Family Member Incarceration	-.11*** [-.17, -.06]	-.14*** [-.21, -.08]	-.04 [-.10, .02]	-.10*** [-.16, -.05]	-.08** [-.14, -.03]	-.08* [-.15, -.02]	-.11*** [-.17, -.05]	-.09** [-.15, -.03]	-.09** [-.15, -.03]	-.07* [-.13, -.01]	-.10*** [-.16, -.05]	-.10*** [-.16, -.05]
Family Member Substance Use	-.12*** [-.17, -.07]	-.13*** [-.20, -.07]	-.04 [-.11, .02]	-.10*** [-.16, -.05]	-.10** [-.15, -.04]	-.09** [-.15, -.03]	-.11*** [-.17, -.06]	-.09** [-.15, -.03]	-.10** [-.16, -.04]	-.07* [-.13, -.01]	-.11*** [-.16, -.05]	-.12*** [-.17, -.06]
Family Member Mental Health	-.11*** [-.16, -.06]	-.12*** [-.19, -.06]	-.03 [-.09, .03]	-.09** [-.14, -.03]	-.10** [-.15, -.04]	-.08* [-.14, -.01]	-.10** [-.16, -.05]	-.08* [-.14, -.02]	-.09** [-.15, -.03]	-.06* [-.12, -.00]	-.10** [-.16, -.04]	-.11*** [-.17, -.06]
Parental Loss	-.13*** [-.18, -.07]	-.11*** [-.18, -.05]	-.04 [-.11, .02]	-.08** [-.14, -.03]	-.07* [-.13, -.02]	-.08* [-.14, -.02]	-.10** [-.15, -.04]	-.07* [-.13, -.01]	-.10** [-.16, -.04]	-.07* [-.13, -.01]	-.09** [-.15, -.03]	-.10** [-.16, -.04]
Familial Violence	-.11*** [-.16, -.06]	-.14*** [-.21, -.08]	-.03 [-.09, .04]	-.09** [-.15, -.03]	-.08** [-.14, -.02]	-.08* [-.14, -.01]	-.09** [-.15, -.03]	-.09** [-.15, -.03]	-.09** [-.15, -.03]	-.06 [-.11, .00]	-.10** [-.16, -.04]	-.11*** [-.16, -.05]

Supplemental Table S7. Associations between number of threat-related adversities, deprivation-related adversities, and total prospectively-ascertained ACEs and age-45 brain structure. White columns denote bivariate associations, whereas the grey columns present results from multiple regression models in which both threat and deprivation were included in the model. Standardized regression coefficients (ß) and 95% confidence intervals are presented in the table. Associations significant at p < .05*, p < .01**, and p < .001*** after FDR correction are marked with asterisks.

		Prospective Threat (0-4)		Prospective Deprivation (0-3)		Prospective ACEs
		Threat experiences	Covarying for Deprivation	Deprivation experiences	Covarying for Threat	Cumulative Score (0-4+)
Age-45 Brain Structure	Total Cortical Surface Area	-.11** (-.16, -.05)	-.10** (-.15, -.05)	-.05 (-.11, .00)	-.03 (-.09, .02)	-.12*** (-.17, -.07)
	Average Cortical Thickness	-.07* (-.13, -.00)	-.04 (-.11, .03)	-.14*** (-.21, -.08)	-.13** (-.20, -.07)	-.13*** (-.20, -.06)
	Accumbens Volume	-.07* (-.13, -.01)	-.07* (-.14, -.01)	< -.00 (-.07, .06)	.01 (-.05, .07)	-.03 (-.10, .03)
	Amygdala Volume	-.10** (-.15, -.04)	-.09* (-.14, -.03)	-.06* (-.12, -.01)	-.05 (-.11, .01)	-.10** (-.15, -.04)
	Brain Stem Volume	-.08* (-.14, -.02)	-.07* (-.13, -.01)	-.08* (-.13, -.02)	-.06 (-.12, -.00)	-.09** (-.15, -.03)
	Caudate Volume	-.08* (-.14, -.02)	-.07* (-.13, -.01)	-.06 (-.12, .01)	-.04 (-.11, .02)	-.08* (-.15, -.02)
	Cerebellum Volume	-.11** (-.16, -.05)	-.09* (-.15, -.03)	-.10** (-.16, -.04)	-.08* (-.14, -.02)	-.10** (-.16, -.04)
	Hippocampus Volume	-.07* (-.13, -.01)	-.06 (-.12, -.00)	-.07* (-.13, -.01)	-.06 (-.12, .00)	-.09** (-.14, -.03)
	Pallidum Volume	-.08* (-.14, -.02)	-.06* (-.13, -.00)	-.07* (-.13, -.01)	-.06 (-.12, .00)	-.10** (-.16, -.04)
	Putamen Volume	-.08* (-.14, -.02)	-.07* (-.13, -.01)	-.04 (-.09, .02)	-.02 (-.08, .04)	-.07* (-.13, -.01)
	Thalamus Volume	-.09** (-.15, -.03)	-.08* (-.13, -.02)	-.08* (-.14, -.02)	-.07 (-.12, -.01)	-.10** (-.16, -.05)
	Ventral Diencephalon Volume	-.10** (-.15, -.04)	-.08* (-.14, -.03)	-.08* (-.14, -.03)	-.07 (-.12, -.01)	-.11*** (-.17, -.05)
